# Interfering with Rac1-activation during neonatal monocyte-macrophage differentiation influences the inflammatory responses of M1 macrophages

**DOI:** 10.1038/s41419-023-06150-y

**Published:** 2023-09-21

**Authors:** Hang Fu, Ping Zhang, Xiao-Dong Zhao, Xiao-Yun Zhong

**Affiliations:** 1 Department of Pediatrics, Women and Children’s Hospital of Chongqing Medical University, 401147 Chongqing, China; 2 Department of Pediatrics, Chongqing Health Center for Women and Children, 401147 Chongqing, China; 3 Chongqing Research Center for Prevention & Control of Maternal and Child Diseases and Public Health, 401147 Chongqing, China; 4 Department of Obstetrics and Gynecology, Women and Children’s Hospital of Chongqing Medical University, 401147 Chongqing, China; 5 Department of Obstetrics and Gynecology, Chongqing Health Center for Women and Children, 401147 Chongqing, China; 6 Chongqing Key Laboratory of Child Infection and Immunity, Ministry of Education Key Laboratory of Child Development and Disorders, National Clinical Research Center for Child Health and Disorders, China International Science and Technology Cooperation Base of Child Development and Critical Disorders, Children’s Hospital of Chongqing Medical University, 400014 Chongqing, China; 7 Department of Rheumatology and Immunology, Children’s Hospital of Chongqing Medical University, 400014 Chongqing, China

**Keywords:** Inflammation, Cytokines

## Abstract

Necrotizing enterocolitis (NEC) is a life-threatening, inflammatory disease affecting premature infants with intestinal necrosis, but the mechanism remains unclear. Neonatal macrophages are thought to play an important role in the pathogenesis of NEC through the production of proinflammatory cytokines. Restriction of cytokine expression in macrophages of NEC tissues may be beneficial. In adult macrophages, interfering with Rac1 has been shown to influence the expression of cytokines. Here, we investigated whether interfering with Rac1 in neonatal macrophages affects their inflammatory responses. First, we found that Rac1-activation was upregulated in the macrophages of rats with NEC model induction compared to controls. The M1 macrophages derived from human neonatal monocytes showed greater Rac1-activation than the M2 macrophages derived from the same monocytes. Inhibition of Rac1-activation by NSC23766 potently reduced the production of proinflammatory cytokines in these M1 macrophages. While neonatal monocytes differentiated into M1 macrophages in vitro, NSC23766 significantly altered cell function during the first six days of incubation with GM-CSF rather than during the subsequent stimulation phase. However, the same effect of NSC23766 was not observed in adult macrophages. Using mass spectrometry, Y-box binding protein 1 (YB1) was identified as being downregulated upon inhibition of Rac1-activation in the neonatal macrophages. Moreover, we found that inhibition of Rac1-activation shortens the poly A tail of PABPC1 mRNA, thereby reducing the translation of PABPC1 mRNA. Consequently, the downregulation of PABPC1 resulted in a reduced translation of YB1 mRNA. Furthermore, we found that TLR4 expression was downregulated in neonatal macrophages, while YB1 expression was reduced. Adding resatorvid (TLR4 signaling inhibitor) to the macrophages treated with NSC23766 did not further reduce the cytokine expression. These findings reveal a novel Rac1-mediated pathway to inhibit cytokine expression in neonatal M1 macrophages and suggest potential targets for the prevention or treatment of NEC.

## Introduction

Necrotizing enterocolitis (NEC) is a severe inflammatory disease that is the most common cause of gastrointestinal mortality in newborns [1]. Premature infants are most at risk for NEC, which has a mortality rate of 20 to 50% [2]. Infants with NEC receive treatment with medication or surgery; however, there is no specific treatment available at present. Many NEC survivors suffer from postoperative complications, such as wound infections, breakdowns or dehiscence, as well as long-term complications, such as intestinal stricture or short-gut syndrome [3]. The pathogenesis of NEC is not fully understood; however, several factors are thought to contribute to its pathogenesis, including prematurity, microbial immaturity, intestinal barrier structure, and the inflammatory response of the premature intestine [4]. Several studies suggest that activation of macrophages contributes to NEC [5–7], and animal and human NEC tissues exhibit a macrophage-rich infiltrate [5, 8]. These intestinal macrophages of NEC tissues display a phenotype similar to M1 macrophages, which produce proinflammatory cytokines [9].

RAS-related C3 botulinum toxin substrate 1 (Rac1) belongs to the Rac family of GTPases, a subfamily of Rho proteins. Rac1 is involved in the regulation of cell migration, phagocytosis, inflammatory responses, actin remodeling and gene expression [10]. Our previous study demonstrated that during monocyte-macrophage differentiation, treatment with statins preserves the capacity of cells to produce proinflammatory cytokines by activating Rac1 [11]. A significant reduction in cytokine expression was observed in this study by adding the Rac1-activation inhibitor NSC23766 during the differentiation of monocytes into macrophages. However, we did not further investigate the signaling pathway downstream of Rac1 in this study. Additionally, other studies have suggested that Rac1 plays a role in macrophage inflammation. The expression of IL-6 and TNF-α was lower in macrophages from the mice lacking Rac1 than in those from wild-type mice [12]. Increased expression of proinflammatory cytokines was observed when Rac1 was overactivated [13]. Upon LPS challenge, Rac1 is involved in activating Syk-MyD88, which in turn regulates the inflammatory responses of macrophages [14].

Although studies suggest that Rac1 is involved in macrophage inflammation, it is of note that these studies were conducted using either adult human macrophages or mouse macrophages. Inflammation of neonatal macrophages has not been addressed in any of these studies. The role of Rac1 in the inflammation of neonatal macrophages is not clear. Since macrophages may importantly contribute to the pathogenesis of NEC, reducing the expression of proinflammatory cytokines in macrophages may alleviate the progression of NEC. Herein, we hypothesize that Rac1 is importantly involved in the regulation of inflammatory responses in neonatal macrophages, which may contribute to the pathogenesis of NEC. To address this issue, we first demonstrated that Rac1-activation in macrophages of intestinal tissue from rats with NEC model induction was more potent than that in macrophages from control rats. By inhibiting Rac1-activation during the differentiation of neonatal monocytes into M1 macrophages, the expression of proinflammatory cytokines was reduced. Following the inhibition of Rac1-activation, we identified that the expression of PABPC1 was downregulated, which resulted in reduced expression of YB1. Finally, the downregulation of YB1 led to reduced expression of TLR4, the receptor required for LPS-induced macrophage inflammation.

## Results

### Rac1-activation in macrophages from rats with NEC model induction is greater than that in macrophages from control rats

**Fig. 1:**
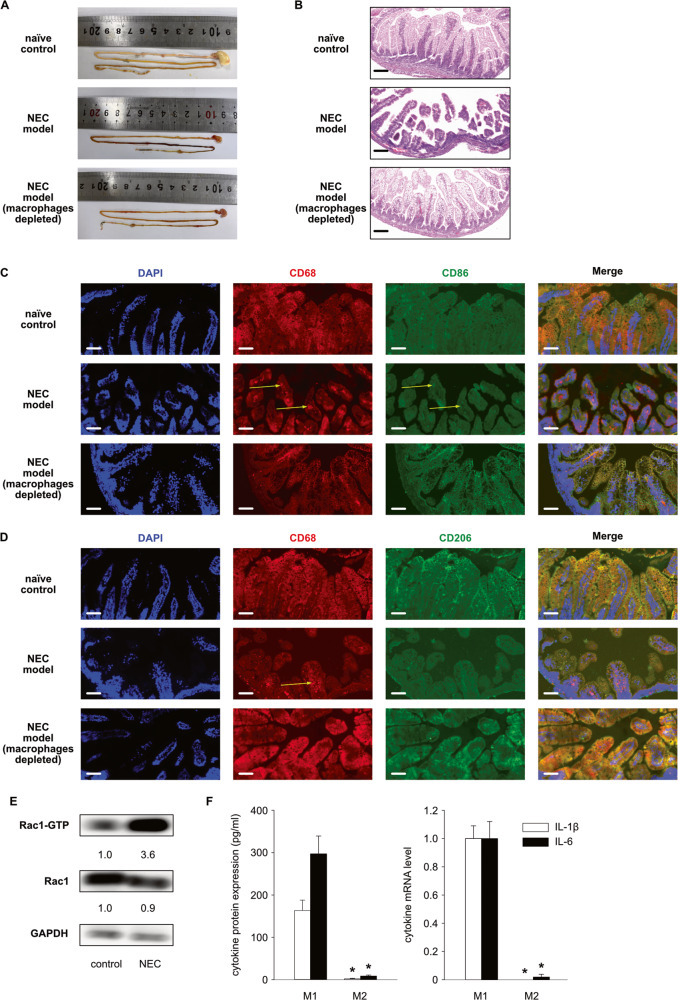
Macrophages from neonatal rats with necrotizing enterocolitis (NEC) display higher Rac1-activation. **A** Representative photographs show intestinal injury in ileal regions in the NEC model of rats. The naïve control and NEC models (macrophages depleted) did not develop intestinal injury. **B** Representative photomicrographs (hematoxylin–eosin; magnification ×20) of the ileum show NEC-like injury in rats. Scale bars = 100 µm. **C** Fluorescence photomicrographs (ileum; magnification ×40) show CD68+CD86+ macrophages (yellow arrow) in the NEC model. DAPI, blue; CD68, red; CD86, green. Scale bars = 50 µm. **D** Fluorescence photomicrographs (ileum; magnification ×40) show CD68+CD206− macrophages (yellow arrow) in the NEC model. DAPI, blue; CD68, red; CD206, green. Scale bars = 50 µm. **E** CD68+ macrophages were isolated and lysed in a cell-lysis buffer provided by the “Active Rac1 Detection Kit”. Pull-down and subsequent Western blot were performed for GAPDH, total Rac1 (Rac1) and activated Rac1 (Rac1-GTP). Values under blots represent the relative protein amounts normalized to GAPDH. **F** Neonatal monocytes from umbilical cord blood were differentiated in vitro into M1 and M2 macrophages. Cells and supernatants were harvested on Day 8. The IL-1β and IL-6 mRNA levels were normalized to HPRT in qPCR. The cytokine concentrations in the supernatants were determined by ELISA. Three additional experiments with similar results were performed. The mean, SD, and significance of these data were calculated in SPSS (Mann–Whitney U test). The asterisks above the lines reflect the significance (*, *p* < 0.05; ns, not significant).

Although macrophage enrichment has been associated with the pathogenesis of NEC, it is not entirely clear how essential these cells are for the progression of the disease. To answer this question, we conducted an animal experiment on rat pups by inducing NEC-like injuries. As part of this experiment, we depleted macrophages in some pups prior to NEC model induction. Thus, the study design included three groups: (1) naïve control; (2) NEC model; and (3) NEC model (macrophages depleted). In contrast to the naïve control, rats of the NEC model developed intestinal injury following 3 days of NEC induction (Fig. 1A, B). In the NEC model (macrophages depleted) group, pups were injected intraperitoneally with clodronate liposomes (50 mg/kg) to deplete macrophages. Depletion of macrophages prevented these pups from developing gastrointestinal injuries. The intestinal crypts from pups of the NEC model showed enrichment of CD68-expressing cells with additional CD86 expression (Fig. 1C) but with less CD206 expression (Fig. 1D), which indicated that these cells are M1-like macrophages. To determine the expression and activation of Rac1 in the macrophages from rats of the naïve control group and the NEC model group, we isolated CD68-expressing cells from intestinal tissue and measured the levels of total Rac1 and Rac1-GTP in these cells. The expression of total Rac1 was similar in both groups, but the expression of Rac1-GTP in the macrophages from rats of the NEC model group was higher than that of the naïve control group (Fig. 1E). These data indicate that Rac1 may be more potently activated in M1 macrophages. Consequently, we investigated whether Rac1-GTP expression was higher in human neonatal M1 macrophages than in M2 macrophages. Monocytes from umbilical cord blood were differentiated into M1 and M2 macrophages in vitro. Because the differentiation protocol is most commonly used for the differentiation of adult macrophages, we first examined the expression of cytokines in our in vitro differentiated M1 and M2 macrophages. As expected, the M1 macrophages showed higher expression of IL-1β and IL-6 at both the protein and mRNA levels (Fig. 1F).

### Inhibition of Rac1-activation alters cells during the first 6 days of in vitro monocyte-macrophage differentiation

**Fig. 2:**
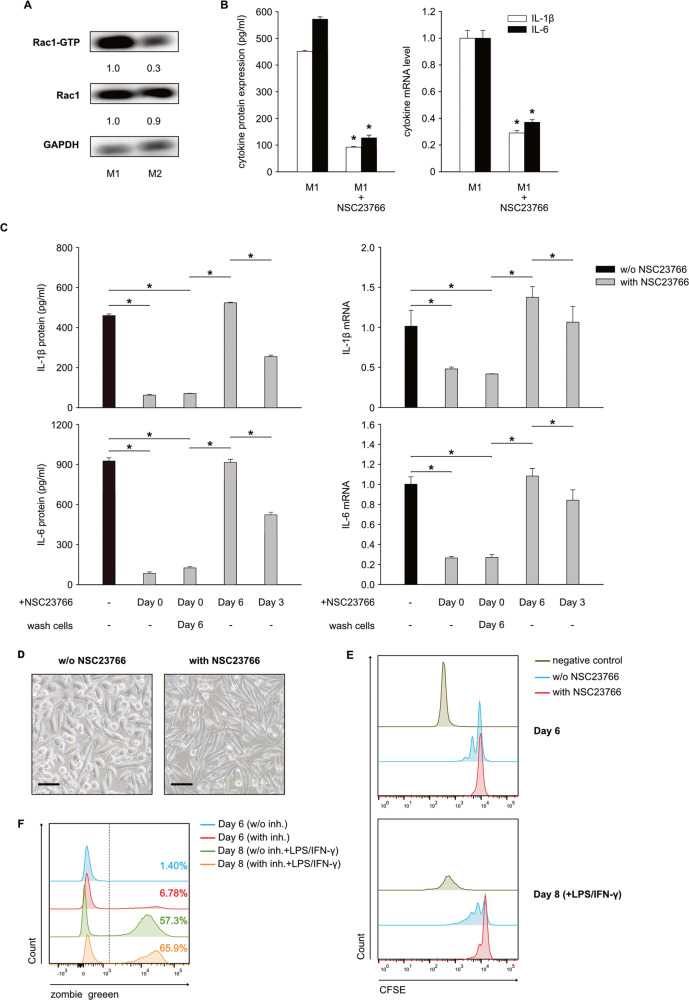
Inhibition of Rac1-activation significantly alters the cell function of neonatal M1 macrophages. **A** Neonatal monocytes from umbilical cord blood were differentiated in vitro into M1 and M2 macrophages. Cells were harvested and lysed in cell-lysis buffer provided by the “Active Rac1 Detection Kit”. Pull-down and subsequent Western blot were performed for GAPDH, total Rac1 (Rac1) and activated Rac1 (Rac1-GTP). Values under blots represent the relative protein amounts normalized to GAPDH. **B** Neonatal monocytes from umbilical cord blood were differentiated in vitro into M1 macrophages with or without NSC23766 (inhibitor of Rac1-activation). Cells and supernatants were harvested on Day 8. The IL1β and IL-6 mRNA levels were normalized to HPRT in qPCR. The cytokine concentrations in the supernatants were determined by ELISA. Three additional experiments with similar results were performed. Data analysis as in Fig. 1F. **C** Neonatal monocytes from umbilical cord blood were differentiated in vitro into M1 macrophages (black column). By adding NSC23766 to the cell culture on different days and washing cells to remove the inhibitor, the presence of this inhibitor was limited in different incubation phases (gray columns). Cells and supernatants were harvested on Day 8. The IL1β and IL-6 mRNA levels were normalized to HPRT in qPCR. The cytokine concentrations in the supernatants were determined by ELISA. Two additional experiments with similar results were performed. Data analysis as in Fig. 1F. **D** Representative photomicrographs of the in vitro differentiated M1 macrophages on Day 6 show differential morphology of cells with or without NSC23766. Scale bars = 50 µm. **E** Representative histograms show proliferation of the in vitro differentiated M1 macrophages on Day 6 and Day 8 with (blue) or without (red) NSC23766. Freshly isolated neonatal monocytes were stained with CFSE (5 µM), and the proliferation of cells was assessed by flow cytometry. The negative control represents unstained cells. **F** Representative histograms show the cell mortality of the in vitro-differentiated M1 macrophages on Day 6 and Day 8 with or without NSC23766. Macrophages were collected on Day 6 and Day 8 and then stained with zombie green. The mortality of cells was assessed by flow cytometry. Blue line, macrophages without NSC23766, Day 6; red line, macrophages with NSC23766, Day 6; green line, macrophages without NSC23766, Day 8 after stimulation; orange line, macrophages with NSC23766, Day 8 after stimulation. Numbers indicate percentages of zombie green + cells.

In the in vitro differentiated M1 and M2 macrophages from neonatal monocytes, the expression of total Rac1 was similar, but the expression of Rac1-GTP was higher in the M1 macrophages (Fig. 2A). We therefore asked whether inhibiting Rac1-activation in M1 macrophages has any effect on cell function. We added NSC237666 to the monocyte cell culture to inhibit the activation of Rac1. NSC23766 significantly inhibited IL-1β and IL-6 expression in M1 macrophages (Fig. 2B). Since the differentiation of monocytes into macrophages undergoes two phases (phase I: 6 days incubation with GM-CSF; phase II: 2 days stimulation with LPS and IFN-γ), we restricted the presence of NSC23766 to just a single phase to identify the drug that exerts the inhibitory effect in either phase I or phase II. NSC23766 was required to inhibit cytokine expression in phase I (Fig. 2C). However, adding the inhibitor in phase II did not inhibit cytokine expression. In addition, we added the inhibitor on the third day of phase I, and this only partially inhibited cytokine expression (Fig. 2C). These data indicated that NSC23766 exerts its function during the first 6 days of incubation. Further investigation was conducted to determine whether NSC23766 affects other aspects of cell function. The morphology of the cells was clearly altered by NSC23766, and these cells became slender in comparison to the M1 macrophages without the inhibitor (Fig. 2D). Cell proliferation was reduced by NSC23766 during phase I (Fig. 2E); however, cell mortality was not significantly altered by this inhibitor (Fig. 2F).

Taken together, these data indicated that inhibition of Rac1-activation potently altered cells during the first 6 days of in vitro monocyte-macrophage differentiation, which further led to a reduction in cytokine expression in these macrophages.

### Inhibition of Rac1-activation reduces the expression of YB1

**Fig. 3:**
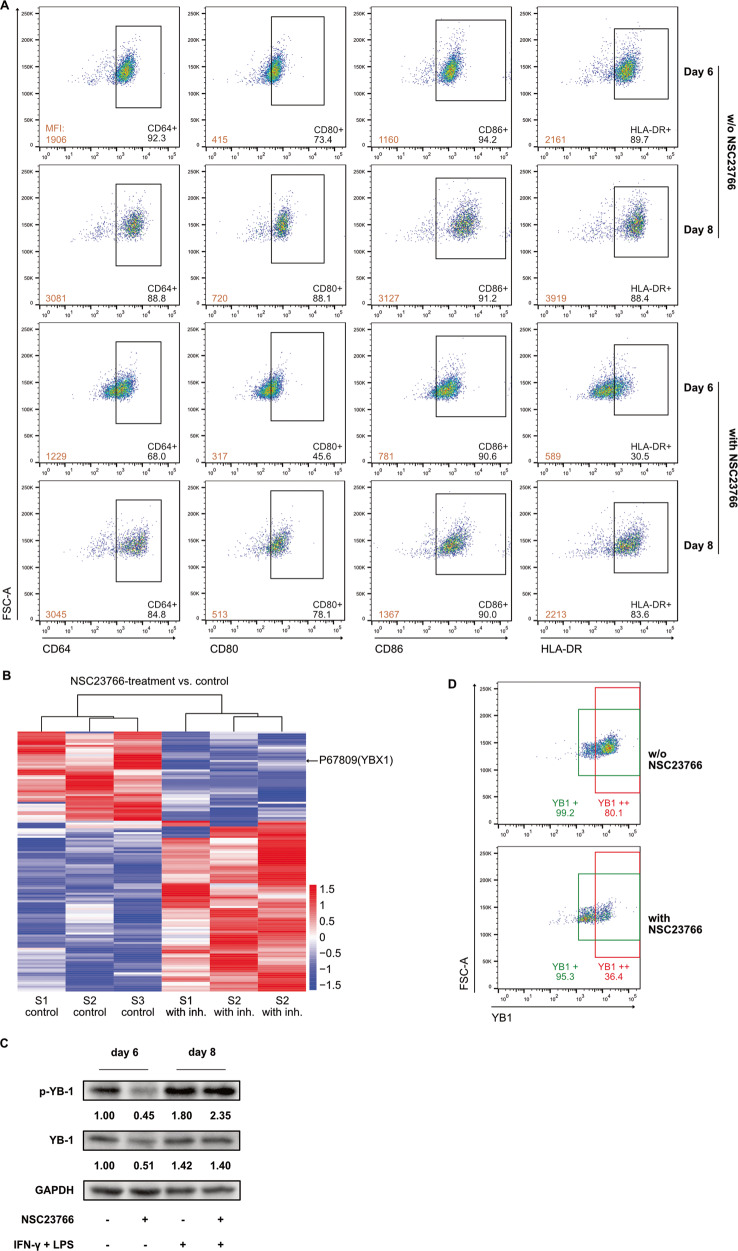
Inhibition of Rac1-activation potently downregulates the expression of YB1 in macrophages. **A** Representative dot plots show differential expression of surface markers (M1 macrophages) of the in vitro differentiated M1 macrophages on Day 6 and Day 8 with or without NSC23766. Macrophages were collected on Day 6 and Day 8 and then stained with anti-CD64, CD80, CD86 and HLA-DR antibodies. The expression of these markers was assessed by flow cytometry. Numbers indicate percentages of protein-expressing cells. **B** Neonatal monocytes from umbilical cord blood were differentiated in vitro into M1 macrophages with or without NSC23766. Cells were collected and lysed on Day 6. Cell lysates were analyzed by label-free mass spectrometry in three independent experiments (S1, S2 and S3), and the differential expression of proteins was analyzed in R (R-3.4.3). Blue and red represent low and high relative expressions, respectively. Refer to Supplementary Table S3 for complete data. **C** Neonatal monocytes from umbilical cord blood were differentiated in vitro into M1 macrophages with or without NSC23766. Cells were collected and lysed on Day 6 and on Day 8. Western blot was performed for GAPDH, total YB1 (YB1) and phosphorylated YB1 (p-YB1). Values under blots represent the relative protein amounts normalized to GAPDH. **D** Representative dot plots show differential expression of YB1 in the in vitro differentiated M1 macrophages on Day 6 with or without NSC23766. Macrophages were collected on Day 6 and stained with anti-YB1 antibody. The expression of this protein was assessed by flow cytometry. YB1-expressing cells (YB1+) are shown in green gates; YB1-high-expressing cells (YB1 ++) are shown in red gates. Numbers indicate percentages of YB1-expressing cells or YB1-high-expressing cells, respectively.

As the inhibition of Rac1 in M1 macrophages significantly altered the expression of cytokines in these cells, it was questionable to consider them to be M1 macrophages. Therefore, surface markers of macrophages with or without NSC23766 were compared. Normally, surface markers of M1 macrophages, such as CD64, CD80 and CD86, are analyzed on Day 8, the day following 2 days of stimulation with LPS and IFN-γ. Nevertheless, our data above showed that NSC23766 altered cells during the 6-day incubation with GM-CSF. Therefore, surface markers of macrophages on both Day 6 and Day 8 were analyzed in our experiments. The expression of CD64, CD80, CD86, and HLA-DR was potently downregulated by NSC23766 on Day 6 and Day 8 (Fig. 3A). A greater amount of inhibition was observed on Day 6 than on Day 8, and all of these surface markers were upregulated after stimulation with LPS or IFN. Meanwhile, CD206 and CD163, which are commonly referred to as markers for M2 macrophages, were not consistently up- or downregulated by NSC23766 (Supplementary Fig. S1A). Next, we sought to identify the signaling pathway downstream Rac1-activation inhibition. Proteome profiling analysis of the cells on Day 6 after treatment with or without NSC23766 revealed that inhibition of Rac1-activation significantly altered cell function and over 100 proteins were differentially expressed (Fig. 3B, Supplementary Fig. S2). One of these proteins was Y-box binding protein 1 (YB1), which may be involved in this regulatory process caused by inhibiting Rac1-activation. YB1 was identified as our target since the literature showed that YB1 expression is associated with macrophage cytokine expression [15, 16]. On Day 6 and on Day 8, Western blot analysis confirmed that YB1 expression and phosphorylated YB1 were downregulated (Fig. 3C). In comparison with Day 6, the expression of YB1 was increased on Day 8, and no clear difference was observed between cells treated with or without NSC23766. In flow cytometry, we found that cells treated with or without NSC23766 both expressed YB-1 (YB1 +, Fig. 3D). However, the number of YB1-high-expressing cells (YB1 ++) was substantially reduced (80.1% vs. 36.4%) by the inhibitor.

### Expression of YB1 is differentially regulated by inhibition of Rac1-activation in neonatal or adult macrophages

**Fig. 4:**
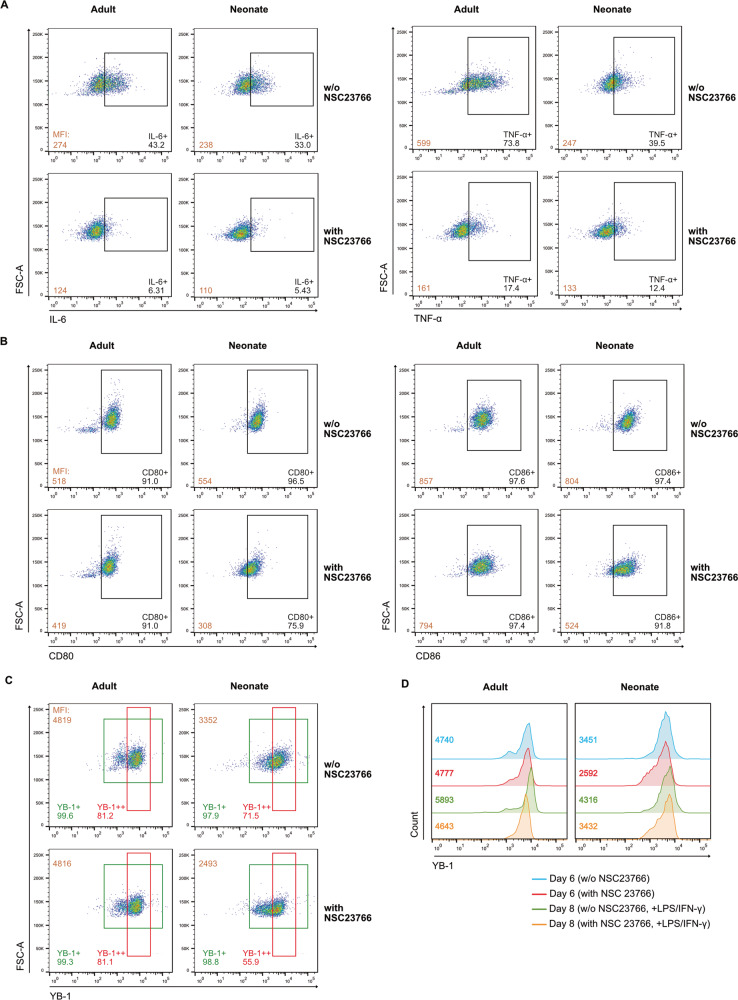
Inhibition of Rac1-activation alters neonatal macrophages differently from adult macrophages. **A** Representative dot plots show differential expression of IL-6 and TNF-α in in vitro-differentiated M1 macrophages from neonates and adults with or without NSC23766. Monocytes from umbilical cord blood or adult peripheral blood were differentiated in vitro into M1 macrophages with or without NSC23766. Cytokine secretion was blocked by Brefeldin A (10 µM), and macrophages were collected on Day 8 and then stained with anti-IL-6 and TNF-α antibodies. The expression of cytokines was assessed by flow cytometry. Numbers in the bottom left indicate the mean fluorescence intensity (MFI) of IL-6 or TNF-α in cells. Numbers in the bottom right indicate percentages of cytokine-expressing cells. **B** Representative dot plots show differential expression of CD80 and CD86 in in vitro-differentiated M1 macrophages from neonates and adults with or without NSC23766. Macrophages were collected on Day 6 and then stained with anti-CD80 and anti-CD86 antibodies. The expression of surface markers was assessed by flow cytometry. Numbers in the bottom left indicate the mean fluorescence intensity (MFI) of CD80 or CD86 in cells. Numbers in the bottom right indicate percentages of protein-expressing cells. **C** Representative dot plots show differential expression of YB1 in in vitro-differentiated M1 macrophages from neonates and adults with or without NSC23766. Macrophages were collected on Day 6 and then stained with an anti-YB1 antibody. The expression of YB1 was assessed by flow cytometry. Numbers in the top left indicate the mean fluorescence intensity (MFI) of YB1 in cells. Numbers at the bottom indicate percentages of YB1-expressing cells (green) or YB1-high-expressing cells (red). **D** Representative histograms show the expression of YB1 in in vitro-differentiated M1 macrophages from neonates and adults with or without NSC23766. Macrophages were collected on Day 6 and Day 8 and then stained with an anti-YB1 antibody. The expression of YB1 was assessed by flow cytometry. Numbers indicate the mean fluorescence intensity (MFI) of YB1 in cells.

As NSC23766 inhibits Rac1-activation in neonatal in vitro differentiated macrophages, we asked whether this effect also occurs in adult macrophages. Similar to neonatal macrophages, inhibition of Rac1-activation potently reduced IL-6 and TNF-α production in adult macrophages (Fig. 4A). Compared to neonatal macrophages, adult macrophages were more capable of producing cytokines (IL-6: 43.2% vs. 33.0%; TNF-α: 73.8% vs. 39.5%). We next investigated expression of surface markers on Day 6 and found that NSC23766 slightly reduced CD80, CD86 and HLA-DR expression in the adult macrophages (Fig. 4B; MFI; CD80: 518 vs. 419; CD86: 857 vs. 794; Supplementary Fig. S1B; MFI; HLA-DR: 903 vs. 873), but was not as potent as in neonatal macrophages (MFI; CD80: 554 vs. 308; CD86: 804 vs. 524; HLA-DR: 1808 vs. 409). In contrast, we found that the expression of CD206 in the adult macrophages was decreased by the inhibitor (Supplementary Fig. S1B), which differs from the neonatal macrophages (Supplementary Fig. S1A). Since the expression of surface markers was differentially regulated by inhibition of Rac1-activation on adult or neonatal macrophages on Day 6, these findings indicate that NSC23766 alters adult macrophages differently from neonatal macrophages. As a result, examining the expression of YB1 in these adult macrophages was also of interest. In contrast to the inhibitory effect observed in neonatal macrophages (Fig. 4C; MFI; 3352 vs. 2493), NSC23766 did not downregulate YB1 expression in adult macrophages on Day 6 (4819 vs. 4816). The expression of YB1 was higher in adult macrophages than in neonatal macrophages. Intriguingly, YB-1 expression was not influenced by the inhibition of Rac1-activation in adult macrophages on Day 6. We next compared the expression of YB1 on both Day 6 and Day 8. Stimulation of the adult macrophages with LPS and IFN-γ upregulated the expression of YB1 (Fig. 4D; MFI; 4740 vs. 5893); however, this upregulation appeared to be blocked in the adult macrophages with NSC23766 (4777 vs. 4643). In contrast, YB1 expression was upregulated following stimulation in neonatal macrophages with or without NSC23766 (w/o NSC23766: 3451 vs. 4316; with NSC23766: 2592 vs. 3432).

In conclusion, these findings suggest that although inhibition of Rac1-activation reduces cytokine expression in both adult and neonatal macrophages, the underlying mechanism may be different.

### Inhibition of Rac1-activation downregulates the expression of YB1, possibly by influencing the expression of PABPC1

**Fig. 5:**
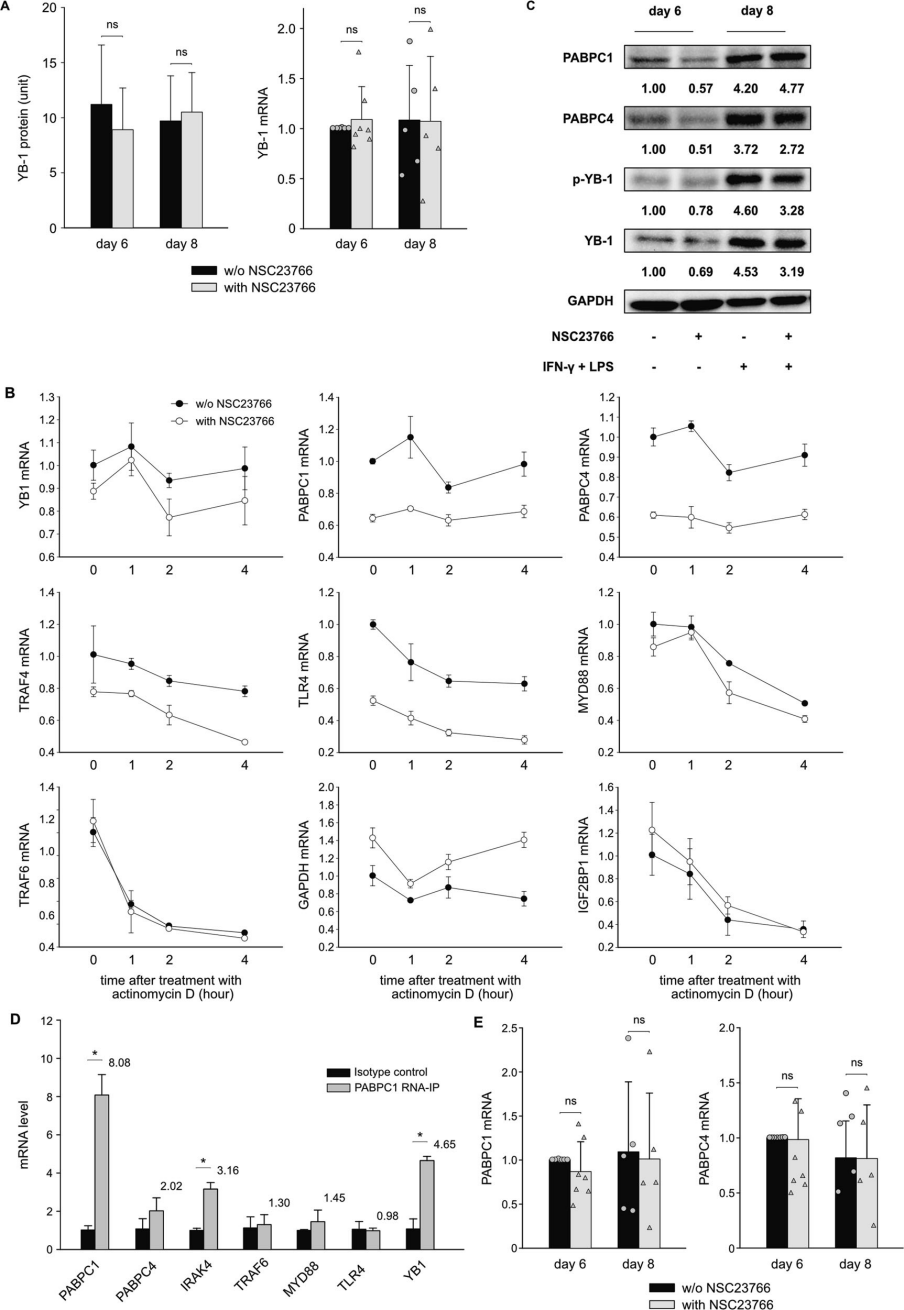
Inhibition of Rac1-activation downregulates the expression of YB1 in neonatal macrophages through inhibition of PABPC1. **A** Neonatal monocytes from umbilical cord blood were differentiated in vitro into M1 macrophages with (gray column) or without (black column) NSC23766. Cells and supernatants were harvested on Day 6 and Day 8. The concentration of YB1 in supernatants was determined using the “PathScan Total YB1 Sandwich ELISA Kit”. Two additional experiments with similar results were performed. Data analysis as in Fig. 1F. The YB1 mRNA level was normalized to HPRT in qPCR. The normalized value of the condition without NSC23766 on Day 6 in each experiment was determined to be 1.0, and the mean ± SD of five to seven experiments was calculated. Data points represent the mRNA level of individual experiments. The mean, SD, and significance of these data were calculated in SPSS (Mann–Whitney U test). The asterisks above the lines reflect the significance (*, *p* < 0.05; ns, not significant). **B** Neonatal monocytes from umbilical cord blood were differentiated in vitro into M1 macrophages with (open circles) or without (filled circles) NSC23766. On Day 6, actinomycin D (5 µg/ml) was added to the cells. Then, cells were collected after 0, 1, 2 and 4 h, and RNA was isolated for RT-qPCR to calculate the relative abundance of mRNA. **C** Neonatal monocytes from umbilical cord blood were differentiated in vitro into M1 macrophages with or without NSC23766. Cells were collected and lysed on Day 6 and on Day 8. Western blot was performed for GAPDH, total YB1 (YB1), phosphorylated YB1 (p-YB1), PABPC1 and PABPC4. Values under blots represent the relative protein amounts normalized to GAPDH. **D** Neonatal monocytes from umbilical cord blood were differentiated in vitro into M1 macrophages. Cells were collected and lysed on Day 6. RNA-IP was performed using isotype control (black columns) or anti-PABPC1 antibody (gray columns). Numbers above the column indicate fold change of PABPC1-binding RNA to isotype control. **E** Neonatal monocytes from umbilical cord blood were differentiated in vitro into M1 macrophages with (gray column) or without (black column) NSC23766. RNA of cells was isolated on Day 6 and Day 8. The mRNA level of PABPC1 or PABPC4 was normalized to that of HPRT in qPCR. The normalized value of the condition without NSC23766 on Day 6 in each experiment was determined to be 1.0, and the mean ± SD of five to seven experiments was calculated. Data points represent the mRNA level of individual experiments. Data analysis as in (**A**).

**Fig. 6:**
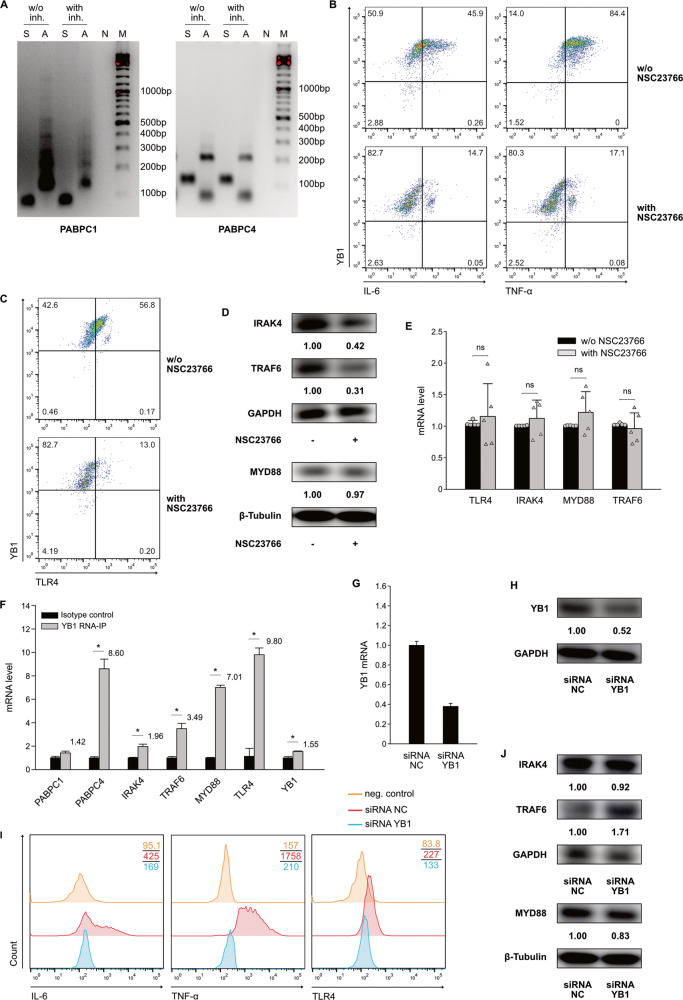
Inhibition of Rac1-activation downregulates the expression of YB1 in neonatal macrophages through inhibition of PABPC1. **A** Neonatal monocytes from umbilical cord blood were differentiated in vitro into M1 macrophages with or without NSC23766. RNA of cells was isolated on Day 6. The length of the poly A tail of PABPC1 and PABPC4 mRNA was detected by using the “USB Poly(A) Tail-Length Assay Kit”. w/o inh., without NSC23766; with inh., with NSC23766; S, gene-specific PCR products of PABPC1 or PABPC4, A, poly A tail PCR products of PABPC1 or PABPC4; N, negative control; M, DNA ladder. **B** Representative dot plots show the relationship of YB1 with cytokine production in in vitro-differentiated M1 macrophages on Day 6 with or without NSC23766. On Day 6, LPS and IFN-γ were added to the cell culture. Meanwhile, cytokine secretion was blocked by adding Brefeldin A (10 µM). After 5 h of incubation, the cells were collected and stained with anti-IL-6, TNF-α and YB1 antibodies. Protein expression was assessed by flow cytometry. Numbers in each quarter of the charts indicate percentages of cells in this quarter. **C** Representative dot plots show the relationship of YB1 with TLR4 expression in in vitro-differentiated M1 macrophages on Day 6 with or without NSC23766. Macrophages were collected on Day 6 and then stained with anti-YB1 and anti-TLR4 antibodies. Protein expression was assessed by flow cytometry. Numbers in each quarter of the charts indicate percentages of cells in this quarter. **D** Neonatal monocytes from umbilical cord blood were differentiated in vitro into M1 macrophages with or without NSC23766. Cells were collected and lysed on Day 6. Western blot was performed for GAPDH, β-Tubulin, MYD88, IRAK4 and TRAF6. Values under blots represent the relative protein amounts normalized to GAPDH or β-Tubulin. **E** Neonatal monocytes from umbilical cord blood were differentiated in vitro into M1 macrophages with (gray column) or without (black column) NSC23766. RNA of cells was isolated on Day 6. The mRNA levels of TLR4, IRAK4, MYD88 or TRAF6 were normalized to HPRT in qPCR. The normalized value of the condition without NSC23766 in each experiment was determined to be 1.0, and the mean ± SD of five experiments was calculated. Data points represent the mRNA level of individual experiments. Data analysis as in Fig. 5A. **F** Neonatal monocytes from umbilical cord blood were differentiated in vitro into M1 macrophages. Cells were collected and lysed on Day 6. RNA-IP was performed using isotype control (black columns) or anti-YB1 antibody (gray columns). Numbers above the column indicate fold change of YB1-binding RNA to isotype control. **G**, **H** Neonatal monocytes were cultured for M1 macrophage differentiation. On Day 2, cells were transfected with siRNA of negative control (siRNA NC) or siRNA YB1. After 48 h, RNA was collected from the cells. After 72 h, the cells were collected and lysed for Western blot. The YB1 mRNA level was normalized to HPRT in qPCR. Data analysis as in Fig. 1F. Western blot was performed for YB1 and GAPDH. Values under blots represent the relative protein amounts normalized to GAPDH. Two additional experiments with similar results were performed. **I** Representative histograms show the expression of cytokines and TLR4 in in vitro-differentiated M1 macrophages from neonates transfected with siRNA NC or siRNA YB1. Macrophages were transfected as described above. On Day 6, cells were collected on Day 6 and then stained with anti-TLR4 antibodies. In parallel, LPS, IFN-γ and Brefeldin A were added to the cell culture. After 5 h of incubation, the cells were collected and stained with anti-IL-6 and anti-TNF-α antibodies. Protein expression was assessed by flow cytometry. Numbers indicate the mean fluorescence intensity (MFI) of protein in cells. **J** Transfected macrophages were prepared as described above. Cells were collected and lysed on Day 6. Western blot was performed for GAPDH, β-Tubulin, MYD88, IRAK4 and TRAF6. Values under blots represent the relative protein amounts normalized to GAPDH or β-Tubulin.

Our data mentioned above showed that inhibition of Rac1-activation downregulated the expression of YB1 in neonatal macrophages on Day 6. The next question was how NSC23766 modulates the expression of YB1 during this process. We first examined YB1 secretion from macrophages with YB1 ELISA on both Day 6 and Day 8 of the cell culture, and there was no significant difference between the culture medium obtained from macrophages with or without NSC23766 (Fig. 5A). We analyzed the mRNA expression of YB1 in these cells and did not find any significant differences. Based on these findings, NSC23766 is believed to inhibit YB1 expression through a posttranscriptional mechanism. We next checked whether NSC23766 influenced mRNA stability. Actinomycin D was added to the cell culture on Day 6 to block the synthesis of new mRNA. NSC23766 did not alter the decay rate of YB1 mRNA or the mRNA of other proteins (Fig. 5B). Then, the expression of PABPC1 and PABPC4, proteins that have been proven to regulate the mRNA translation of YB1 in cells, was analyzed by Western blot. In a manner similar to that observed with YB1, NSC23766 also reduced the expression of PABPC1 and PABPC4 in macrophages on Day 6 (Fig. 5C). To confirm whether PABPC1 and PABPC4 bind to the mRNA of YB1 in our neonatal macrophages, RNA-IP was used to precipitate PABPC1- or PABPC4-binding mRNA. As shown in Fig. 5D, PABPC1 bound to YB1 mRNA as well as to its own mRNA. However, PABPC4 did not bind to YB1 mRNA (Supplementary Fig. S3). PABPC1 and PABPC4 mRNA expression was not significantly altered by NSC23766 (Fig. 5E), and their mRNA decay rate was not affected by NSC23766 (Fig. 5B). These results indicated that NSC23766 downregulates PABPC1 and PABPC4 at the posttranscriptional level. PABPC1 binds to its own mRNA, and the literature suggests that the length of the poly A tail of PABPC1 mRNA may be important for the regulation of PABPC1 mRNA translation [17]. Consequently, we measured the length of the poly A tail of PABPC1 and PABPC4 mRNA in macrophages in the presence or absence of NSC23766. The inhibition of Rac1-activation shortened the poly A tail of PABPC1 but not PABPC4 mRNA in macrophages (Fig. 6A).

Collectively, these findings suggest that inhibition of Rac1-activation during the differentiation of neonatal monocytes to M1 macrophages downregulates PABPC1 expression by shortening the poly A tail of its mRNA, thereby limiting the expression of YB1.

### Downregulation of YB1 in macrophages inhibits the expression of TLR4

Next, we analyzed whether downregulation of YB1 alters the expression of cytokines in M1 macrophages. Flow cytometry analysis showed that IL-6- and TNF-α-producing cells were YB1-high-expressing cells (Fig. 6B). YB1 was downregulated in cells by NSC23766, and these YB1-low-expressing cells were incapable of producing IL-6 or TNF-α. Macrophages were stimulated with LPS, so we investigated whether NSC23766 affected the expression of proteins involved in endotoxin-triggered signaling. NSC23766 potently inhibited the expression of TLR4 on macrophages, and TLR4-expressing cells were YB1-high-expressing cells (Fig. 6C). Western blot analysis showed that NSC23766 inhibited the expression of IRAK4 and TRAF6, but not MYD88 (Fig. 6D). However, NSC23766 did not significantly alter the mRNA expression of TLR4, IRAK4, MYD88, and TRAF6 (Fig. 6E). RNA-IP analysis of YB1 revealed that YB1 bound to the TLR4, MYD88, and TRAF6 mRNAs (Fig. 6F). These data indicated that YB1 may affect the mRNA translation of TLR4, MYD88 and TRAF6. We then transfected YB1-siRNA into the cells to determine whether downregulation of YB1 affects the expression of cytokines and proteins involved in the LPS-triggered signaling pathway. Transfection with YB1-siRNA significantly inhibited the expression of YB1 mRNA and protein (Fig. 6G, H). The expression of IL-6 and TNF-α was reduced by transfection with YB1-siRNA (Fig. 6I; IL-6: 425 vs. 169; TNF-α: 1758 vs. 210). In addition, transfection with YB1-siRNA downregulated the expression of TLR4 in macrophages. Nevertheless, this transfection did not affect the expression of MYD88, IRAK4 or TRAF6 (Fig. 6J).

**Fig. 7:**
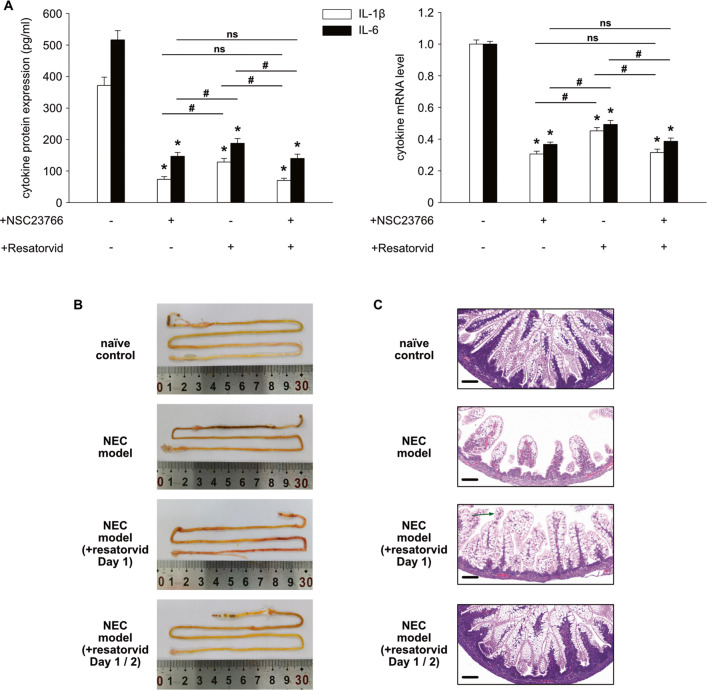
Inhibition of Rac1-activation reduces cytokine expression via downregulation of TLR4. **A** Neonatal monocytes from umbilical cord blood were differentiated in vitro into M1 macrophages. NSC23766 (30 µM) or resatorvid (40 nM) was added to the cell culture as indicated. Resatorvid was added simultaneously with LPS and IFN-γ stimulation. Cells and supernatants were harvested on Day 8. The IL1β and IL-6 mRNA levels were normalized to HPRT in qPCR. The cytokine concentrations in the supernatants were determined by ELISA. Two additional experiments with similar results were performed. The mean, SD, and significance of these data were calculated in SPSS (Mann–Whitney U test). The asterisks above the columns reflect the significance of with inhibitor vs. without inhibitor (*, *p* < 0.05; ns, not significant). The hashes above the lines reflect the significance (#, *p* < 0.05; ns, not significant). **B** Representative photographs show that the addition of resatorvid prevents intestinal injury in ileal regions in the NEC model of rats. Only rats in the NEC model developed severe intestinal injury. Resatorvid (4 mg/kg) was intraperitoneally injected into the rats either only on the first day or on the first and second days of NEC model induction. **C** Representative photomicrographs (hematoxylin–eosin; magnification ×20) of the ileum show NEC-like injury in rats. Scale bars = 100 µm. Green arrow, epithelial cell desquamation.

To confirm that inhibition of Rac1-activation reduces the expression of proinflammatory cytokines in macrophages through downregulation of TLR4, resatorvid, a TLR4 signaling inhibitor, was added to our in vitro culture experiments in addition to LPS and IFN-γ stimulation. As expected, the addition of resatorvid potently reduced IL-1β and IL-6 cytokine expression, although not as potently as NSC23766 (Fig. 7A). A further reduction in cytokine expression was not observed when resatorvid was added to macrophages treated with NSC23766 compared to cells not treated with resatorvid. To further identify the contribution of TLR4 to the pathogenesis of NEC, we injected resatorvid peritoneally into rats with NEC. Resatorvid significantly reduced intestinal tissue damage caused by NEC model induction (Fig. 7B, C). In contrast to the NEC model, injection of resatorvid only on the first day of induction potently prevented intestinal injuries. However, epithelial cell desquamation was observed in the rats of this group (Fig. 7C, green arrow), and the intestinal tissue was still injured. In contrast, following the injection of resatorvid on the first and second days of NEC model induction, intestinal tissues were completely protected from NEC-like injuries. Due to the high abundance of TLR4 in monocytes and macrophages, this result indicates that TLR4 signaling in macrophages may play an important role in the pathogenesis of NEC.

Together, these data suggest that the downregulation of YB1 in macrophages reduces the expression of TLR4, which in turn limits the expression of proinflammatory cytokines.

## Discussion

Previous studies have indicated that macrophages and the excessive production of proinflammatory cytokines they produce are importantly involved in the pathogenesis of NEC [5–8]. These macrophages in intestinal tissues with NEC resemble M1 macrophages [7, 8]. Different physiological conditions allow monocytes to differentiate into M1 or M2 macrophages. In response to IFN-γ or TLR4 ligands (bacterial LPS), M1 macrophages produce large amounts of proinflammatory cytokines, including IL-1β, IL-6 and TNF-α. In contrast, M2 macrophages can be polarized by IL-4, IL-10, IL-13, TGF-β and glucocorticoids and exhibit anti-inflammatory activities by releasing large amounts of IL‐10 and TGF‐β [18]. Because macrophages in intestinal tissues with NEC secrete a high level of proinflammatory cytokines, limiting the release of these cytokines or modifying the phenotype of macrophages may be effective in alleviating the progression of the disease. Our previous studies have demonstrated that statins retain the capacity of macrophages to respond to endotoxin through Rac1-activation [11]. According to this study, Rac1-activation plays a significant role in the differentiation of monocytes into macrophages. Additionally, other studies have demonstrated a role for Rac1-activation in the production of cytokines by macrophages [12–14, 19]. Considering that these studies were conducted on adult macrophages, it is not known whether Rac1 plays a role in neonatal macrophages. Thus, we aimed to investigate how Rac1 plays a role in the inflammation of neonatal macrophages in this study. It is possible that interfering with Rac1 could alter the inflammatory response of neonatal macrophages, which could be beneficial in the progression of NEC.

First, we examined the expression and activation of Rac1 in macrophages of intestinal tissues with and without NEC. In animal experiments, hypothermia and hypoxia were used to induce a model of NEC in rat pups. The intestinal tissue of NEC infants was not investigated in this experiment since a sufficient amount of intestinal tissue could not be obtained as a control. We observed more CD68+CD86+CD206− cells in the crypts of the intestines from the NEC-inducing rats than in those from the control rats. Depletion of macrophages in NEC-inducing rats can ameliorate the progression of NEC. These results suggest that macrophages play an important role in the pathogenesis of NEC. It should be noted that most of the previous publications regarding macrophages in NEC have focused primarily on the infiltration of macrophages in the intestinal tissues of NEC infants or animal models of NEC [5, 6]. In our research, we injected clodronate liposomes into the rats prior to induction of the NEC model. As a result of the removal of macrophages from rat pups, NEC progression is significantly reduced, which underscores the importance of macrophages in the pathogenesis of NEC. Rac1 activation was found to be more potent in macrophages isolated from the intestinal tissues of NEC-inducing rats than in controls. Considering that macrophages from NEC-induced rats exhibit more characteristics of M1 macrophages. We hypothesized that Rac1-activation in M1 macrophages may be greater than that in M2 macrophages. To prove this hypothesis, we isolated monocytes from cord blood and performed in vitro differentiation into M1 and M2 macrophages. In contrast to the differentiation of M2 macrophages, we found that Rac1 is more potently activated in differentiated M1 macrophages. Thus, activation of Rac1 may play a critical role in the differentiation of monocytes into macrophages. Furthermore, we found that inhibition of Rac1-activation potently reduced the expression of proinflammatory cytokines in M1 macrophages. As a point of clarification, the in vitro differentiation protocol we used in this study is a classical procedure: 6 days of incubation with GM-CSF (differentiation phase) followed by 2 days of stimulation with LPS and IFN-γ (stimulation phase). To reduce cytokine production effectively, Rac1-activation must be inhibited during the differentiation phase but not during the stimulation phase. It appears that Rac1-activation is required during the early phase of neonatal monocyte-macrophage differentiation but not during LPS- and IFN-γ-triggered inflammation. This result may suggest that Rac1 has a different function in neonatal macrophages than it does in adult macrophages. Previous studies revealed that PI3K, JNK and p38-MAPK are downstream of Rac1-activation [20–23], and these proteins are essential in LPS- or IFN-γ-triggered inflammation in macrophages [24–26]. Our further investigation focused on the changes in M1 macrophages that are caused by the inhibition of Rac1 activation. The morphology and proliferation of macrophages are influenced by this inhibition. Because only some macrophages proliferate during monocyte-macrophage differentiation, altering proliferation is unlikely to have a significant impact on cytokine production. The inhibitor had no effect on the mortality of cells. Based on these results, it appears that the inhibitory effect of cytokine expression in M1 macrophages does not result from changes in the number of cells in the cell culture. The term M1 macrophages refers to macrophages on Day 8 (after both the differentiation and stimulation phases), and in our study, Rac1-activation was necessary in the differentiation phase. It is therefore important to determine the status of macrophages on Day 6. M1 macrophages are thought to be distinguished by certain surface markers, such as CD64, CD80, CD86, and HLA-DR [18, 27]. The surface markers for M1 macrophages were downregulated by inhibition of Rac1-activation on both Day 6 and Day 8. However, the inhibition did not result in increased expression of CD206 and CD163, which are surface markers for M2 macrophages. Based on this result, we conclude that inhibition of Rac1-activation does not shift the differentiation from M1 to M2 macrophages, and this inhibition probably alters macrophages during the first 6 days of incubation.

As part of our efforts to understand the signaling pathway downstream of Rac1-activation, we conducted proteome profiling analysis in macrophages on Day 6. In this study, the cold shock protein YB1 was identified, and its expression was reduced following the inhibition of Rac1-activation. As a DNA/RNA binding protein, YB1 plays a role in many cellular processes, including DNA transcription, splicing of pre-mRNA, translation of mRNA, drug resistance, and DNA repair [28, 29]. Since the YB1 protein is capable of binding both DNA and mRNA, thereby modulating transcription and translation, the location of YB1 may be crucial for its function. The phosphorylation of YB1 at Ser102 promotes its translocation from the cytoplasm to the nucleus [30]. YB1 may participate in regulating inflammation in macrophages, and the expression of IL-1β, IL-6 and CCL5 has been found to be regulated by YB1 in cells [15, 16, 31].

The inhibition of Rac1-activation modulates the differentiation of neonatal monocytes into macrophages. Furthermore, we investigated whether this inhibition will have the same effect on adult macrophages as well. As a result of inhibiting Rac1-activation in adult M1 macrophages, cytokine expression was reduced; however, the expression of surface markers for M1 macrophages CD80, CD86 and HLA-DR was not potently affected on Day 6. In line with the surface marker, the expression of YB1 was not reduced on Day 6. Nevertheless, we found that YB1 expression is upregulated in response to stimulation with LPS and IFN-γ and that this upregulation was prevented by inhibiting Rac1-activation. The upregulation of YB1 in macrophages following LPS stimulation was consistent with previous data [16, 32]. It appears that neonatal macrophages express YB1 differently than adult macrophages when Rac1-activation is inhibited. According to these results, although inhibiting Rac1-activation reduces the expression of proinflammatory cytokines in both neonatal and adult macrophages, the signaling pathway involved is different in each case.

The expression of YB1 on Day 6 was reduced by inhibition of Rac1-activation. It is fascinating to see how Rac1 influences the expression of YB1 during differentiation. First, we investigated whether inhibiting Rac1-activation promotes YB1 secretion because YB1 has been reported to be secreted by immune cells following inflammatory challenges [16, 33]. The secretion of YB1 was not affected by this inhibition. The mRNA data of YB1 indicate that the protein is modulated at the posttranscriptional level. By adding actinomycin D to the cell culture, we found that the stability of YB1 mRNA, as well as the mRNA of other proteins, was not affected by inhibition of Rac1-activation. As a result of inhibition of Rac1 activation, the expression of the PABPC1 and PABPC4 proteins, which have been implicated in regulating the translation of YB1 mRNA [34], was downregulated. PABPC1 bound to the mRNA of YB1 in our RNA-IP experiments, further supporting the hypothesis that PABPC1 regulates the translation of YB1 mRNA. Consequently, we asked how inhibition of Rac1-activation affected the expression of PABPC1 in macrophages. As a result of the inhibition, neither the expression of mRNA for PABPC1 nor the stability of PABPC1 were altered. Thus, inhibition of Rac1-activation appears to downregulate PABPC1 expression at the translational level. According to research, the length of the poly A tail of PABPC1 mRNA is crucial for translation [17]. Therefore, the length of the poly A tail of PABPC1 mRNA was assessed in macrophages with or without the inhibitor on Day 6. The inhibition of Rac1-activation resulted in the shortening of the poly A tail of PABPC1 mRNA. These results suggest that inhibition of Rac1-activation reduces PABPC1 expression by changing the length of the poly A tail of the mRNA, and this reduction in PABPC1 further limits the translation of YB1 mRNA. We have demonstrated that the expression of YB1 was downregulated by inhibition of Rac1-activation, and the next question is whether altering the expression of YB1 impacts cytokine expression in neonatal M1 macrophages. We first found that IL-6- or TNF-α-producing cells were YB1-high-expressing cells. Since M1 macrophages were stimulated by LPS and IFN-γ, we investigated whether the proteins involved in the LPS-triggered signaling pathway are downstream of YB1 in macrophages. We found that YB1 binds to the mRNA of TRAF6, MYD88 and TLR4. Knockdown of YB1 in macrophages downregulates the expression of TLR4 on macrophages; however, the expression of TRAF6 and MYD88 was not altered by knockdown of YB1. The receptor TLR4 is required for the initiation of inflammatory responses in macrophages triggered by LPS [35]. This result indicates that YB1 affects the expression of TLR4 during the differentiation of neonatal monocytes into M1 macrophages, which further affects macrophage cytokine production. Next, we used the TLR4 signaling inhibitor resatorvid in cell culture to confirm that inhibition of Rac1-activation resulted in a reduction in macrophage cytokine expression due to the downregulation of TLR4. Our results suggest that the downregulation of TLR4 is indeed a significant contributor to this effect and that inhibition of Rac1-activation may exert other effects independent of TLR4. The animal experiment with resatorvid demonstrated that TLR4 signaling plays a crucial role in the pathogenesis of NEC. The intestinal macrophages express a high level of TLR4; however, the intestinal epithelial cells of neonates, particularly premature neonates, also express a high level of TLR4 [36]. Thus, it is not completely clear whether the inhibition of TLR4 signaling primarily affects macrophages or epithelial cells. By inhibiting TLR4 signaling, it is possible to have an effect on both macrophages and epithelial cells in the pathogenesis of NEC. Since our findings indicate that inhibition of Rac1-activation results in the downregulation of TLR4 in macrophages, it would be interesting to determine whether epithelial cells have the same regulation in the future.

In conclusion, our study demonstrated that Rac1-activation was greater in macrophages from NEC-inducing rats than in macrophages from control rats. During the in vitro differentiation of neonatal monocytes into M1 macrophages, inhibition of Rac1-activation reduced the expression of proinflammatory cytokines in macrophages by interfering with the Rac1-PABPC1-YB1-TLR4 signaling pathway. Inhibiting Rac1-activation in macrophages may be a potential therapeutic target in the prevention or treatment of NEC.

## Materials and methods

### Materials

The materials used are presented in the respective text. The antibodies used in this study are listed in Supplementary Table 1. The sequences of the primers and siRNAs used in this study are listed in Supplementary Table 2.

### Monocyte isolation

Mononuclear cells (MNCs) were isolated from umbilical cord blood, which was collected by the midwife in the delivery room. Written informed consent was signed by the parents of the infants. The use of the cells was approved by the local ethical committee. The cord blood was mixed with the same volume of DPBS (Biosharp, Hefei, China) and subjected to gradient centrifugation using Lymphoprep (15 min, 1200×*g*; STEMCELL Technologies, Shanghai, China) in a SepMate-50 (RUO) tube (STEMCELL Technologies). The obtained cells were washed twice (250×*g*, 10 min) in DPBS, containing 2% BSA (Absin Bioscience, Shanghai, China). Monocytes were prepared from the MNC using the “EasySep Human CD14 Positive Selection Kit II” (STEMCELL Technologies).

### Cell culture

To obtain M1 macrophages, freshly isolated monocytes were incubated for 6 days in X-VIVO 15 medium (Lonza, Guangzhou, China) with GM-CSF(100 ng/ml, Abmole Bioscience, Houston, USA). On Day 6, LPS (10 ng/ml, Sigma-Aldrich, Shanghai, China) and IFN-γ (50 ng/ml, Sino Biological, Beijing, China) were added, and the cells were then incubated for an additional 2 days. To obtain M2 macrophages, freshly isolated monocytes were incubated for 6 days in X-VIVO 15 medium (Lonza, Guangzhou, China) with M-CSF (100 ng/ml, Sino Biological). On Day 6, IL-4, IL-10 and TGF-β (20 ng/ml, Sino Biological) were added, and the cells were further incubated for 2 days. In some experiments, the Rac1 inhibitor NSC23766 (30 µM, Abmole Bioscience) was added to the freshly isolated monocytes and incubated for 8 days with cells. In some experiments, the TLR4 signaling inhibitor resatorvid (40 nM, Abmole Bioscience) was added to the cell culture on Day 6.

### Rat NEC model

Animal studies were conducted following ethical approval from the Institutional Animal Care and Use Committees at Cloud-Clone Animal incorporated and complied with all relevant ethical regulations for animal research. Fifteen neonatal SD rats were randomly assigned to three study groups: (1) naïve control; (2) NEC model; and (3) NEC model (macrophages depleted). The sample size was determined by estimating the intestinal injury in our preliminary experiments. The pups of the naïve control were maintained without intervention. The pups of the NEC model were fed rat milk Formula 6 times per day with an increasing caloric intake every day for 3 days. The pups were treated twice a day with hypoxia (5% oxygen + 95% nitrogen, 10 min) and hypothermia (4 °C, 10 min). The pups of the NEC model (macrophages depleted) received peritoneal injection of “clondronate liposomes” (50 mg/kg; Liposoma, Amsterdam, Netherlands) 2 days prior to the identical treatment of the NEC model as the group b. All pups were sacrificed on Day 4. The complete intestine of the pups was imaged, and the terminal ileum was collected for HE staining and immunofluorescence staining. The rest of the intestinal tissue was prepared for isolating macrophages. In the other animal experiments, the TLR4 signaling inhibitor resatorvid (4 mg/kg, Abmole Bioscience) was intraperitoneally injected into the rats with NEC model induction either only on the first day of induction or on the first and second days of induction.

### Macrophage isolation

Intestinal tissue was washed with Hank’s balanced salt solution (HBSS, Biosharp) and then treated first with HBSS containing 1 mM EDTA (Sigma-Aldrich) for 20 min at 37 °C, followed by collagenase type IV (STEMCELL Technologies) for 2 h at 37 °C. The cell suspension was incubated at 4 °C for 30 min with an anti-rat CD68 antibody (biotinylated, Supplementary Table 1). Afterward, MojoSort Streptavidin Nanobeads (BioLegend, San Diego, CA, USA) were added to the cell suspension and incubated at 4 °C for 15 min. CD68+ cells were isolated by magnetic cell sorting.

### ELISA cytokine analysis

ELISAs (ELISA for IL-1β or IL-6) were performed according to the manufacturer’s instructions (Elabscience Biotechnology, Wuhan, China). The secretion of YB1 by macrophages was detected by the “PathScan Total YB1 Sandwich ELISA Kit” (Cell Signaling Technology, Shanghai, China) following the manufacturer’s instructions.

### Active Rac1 detection assay

To analyze Rac1-activation, cells were washed with PBS and lysed using cell lysis buffer provided by the “Active Rac1 Detection Kit” (Cell Signaling Technology). Protein concentration was determined using the “Enhanced BCA Protein Assay Kit” (Beyotime Biotechnology, Shanghai, China). Then, the cell lysates were incubated at 4 °C with the GST-PAK1-PBD and glutathione resin for 1 h on a rotator. The unbound protein was removed by washing with lysis/binding/wash Buffer and centrifugation (1 min, 4 °C, 5000 × *g*), and then the binding GTPase was detached by adding SDS sample buffer to the glutathione resin. The detached GTPase was analyzed by Western blot.

### Western blot

Samples were incubated for 8 min at 95 °C in “SDS-PAGE Sample Loading Buffer (5X)” (Beyotime Biotechnology). Proteins were separated using standard SDS-PAGE (12% or 10%) and electroblotted (1 mA/cm^2^, 1.5 h) onto a nitrocellulose blotting membrane (0.45 m, Biosharp). The membranes were blocked using 5% BSA in TBST (BSA, fraction V, Biosharp; 1 h, 4 °C; TBST consists of 20 mM Tris-HCl, 150 mM NaCl (pH 7.5) and 0.1% Tween 20). The blots were incubated overnight with the primary antibody (4 °C). The blots were washed three times with TBST, incubated for 1 h at room temperature with the appropriate secondary antibody, and then washed three times again with TBST. Data analysis was performed using BeyoECL Star (ECL like Western reagent) (Beyotime Biotechnology) in the ChemiDoc XRS+ Gel Imaging System (Bio-Rad Laboratories, Shanghai, China). Density analysis was performed using Quantity One software (Bio-Rad Laboratories).

### RNA isolation, reverse transcription and quantitative PCR

Total RNA was extracted with the “SPARKeasy Improved Tissue/Cell RNA Kit” (Sparkjade Biotechnology, Qingdao, China) according to the manufacturer’s instructions. Reverse transcription was performed using the “SPARKscript II RT Plus Kit (With gDNA Eraser) “ (Sparkjade Biotechnology). Real-time PCR was conducted in a volume of 15 µl consisting of 1 µl cDNA, 7.5 µl “2×SYBR Green qPCR Mix” (Sparkjade Biotechnology), 1.5 µl sense primer, 1.5 µl antisense primer (0.25 µM each), and 3.5 µl nuclease-free water. The primers were obtained from Sangon Biotech (Shanghai, China). The sequences of the primers are listed in Supplementary Table 2. HPRT was amplified as an internal reference. Data were analyzed using a comparative ΔΔCT method.

### Liquid chromatography-mass spectrometry (LC-MS)

Label-free LC-MS was conducted in our experiments to identify downstream targets of Rac1-activation. Macrophages were lysed with DB lysis buffer (8 M urea, 100 mM TEAB, pH 8.5). The samples were trypsinized and fractionated using a C18 column (Waters BEH C18, 4.6 × 250 mm, 5 μm; Agilent, Beijing, China) on a Rigol L3000 HPLC system. All fractions were dried under vacuum and then reconstituted in 0.1% (v/v) formic acid (FA) in water. The LC-MS analyses were performed using an EASY-nLC^TM^ 1200 UHPLC system (Thermo Fisher) coupled with a Q Exactive^TM^ HF-X mass spectrometer (Thermo Fisher) at Novogene Co., Ltd. (Beijing, China). All resulting spectra were searched against “gallus_gallus_uniprot_2021_7_15. fasta. fasta (34808 sequences)” database by the search engine: Proteome Discoverer 2.4 (Thermo Fisher). Please contact the corresponding author Hang Fu if you wish to obtain the complete raw data.

### Transfection experiments

On Day 0, freshly isolated monocytes were incubated with GM-CSF. On Day 1, Lipofectamine RNAiMAX Reagent (Invitrogen, Thermo Fisher Scientific, Shanghai, China) and siRNAs (GenePharma, Shanghai, China) were diluted separately in X-VIVO 15 medium. The siRNA sequences are listed in Supplementary Table 2. Equal volumes of diluted transfection reagent were mixed with siRNA and incubated for 5 min at room temperature. Subsequently, 50 µl of the mix was added to 450 µl of cell culture in 48 well plates (Jet Bio-Filtration, Guangzhou, China). The cells were further incubated according to the M1 macrophage culture protocol mentioned above. Cells were collected or cell lysates were prepared at several time points for further analysis by FACS, qPCR, or Western blot.

### Flow cytometry

The macrophages were washed with DPBS and then incubated with Accutase solution (STEMCELL Technologies) for 15 min at 37 °C. Following incubation, macrophages were detached by robust pipetting, and then the cells were collected and washed with PBS/BSA (PBS + 2% BSA). For surface staining, the cells were directly incubated with antibodies against surface markers. For intracellular staining, the cells were fixed with 4% paraformaldehyde fix solution (Beyotime Biotechnology) and permeabilized with immunostaining permeabilization buffer with Triton X-100 (Beyotime Biotechnology). The cells were then incubated with antibodies against intracellular proteins. At least 5000 cells per sample were analyzed. Samples were acquired with a DxP Athena flow cytometer (Cytek Biosciences, Fremont, CA, USA) and analyzed using FlowJo software (BD Biosciences, Franklin Lakes, NJ, USA). Background signals were controlled by applying a fluorescence minus one (FMO) control.

### RNA immunoprecipitation (RNA-IP)

Protein A/G Magnetic Beads (MedChemExpress, Shanghai, China) were washed three times with TBST. The beads were incubated with the antibody in cell lysis buffer (Beyotime Biotechnology) for 20 min at room temperature on a shaker. Following this, yeast RNA (Beyotime Biotechnology) and FBS (AusgeneX, Oxenford, Australia) were added to the beads, and the beads were incubated for another 20 min. The beads were washed three times with TBST. Subsequently, cell lysates were added to the beads and then incubated on a shaker for 30 min at room temperature. Following incubation, the beads were washed three times with TBST. Then, “RLT Plus Lysis Buffer” from the “SPARKeasy Improved Tissue/Cell RNA Kit” (Sparkjade Biotechnology) was added to the beads. The samples were ready for RNA isolation using the “SPARKeasy Improved Tissue/Cell RNA Kit”.

### Poly A tail length detection assay

To detect the length of the poly A tail of mRNAs, “USB Poly(A) Tail-Length Assay Kit” (Affymetrix, Cleveland, Ohio, USA) was used according to the manufacturer’s instructions. The primers used in this assay are listed in Supplementary Table 2.

### RNA decay assay

To evaluate RNA stability in macrophages, actinomycin D (AbMole Bioscience) was added to cells on Day 6 at a final concentration of 5 μg/ml. Then, cells were collected after 0, 1, 2, and 4 h, and RNA was isolated for RT-qPCR to calculate the relative abundance of mRNA.

### HE staining

The terminal ileum was fixed in formalin, embedded in paraffin and sliced into paraffin sections. This section was prepared for subsequent HE staining or immunofluorescence staining. The slides were deparaffinized and then stained with “Hematoxylin and Eosin Staining Kit” (Beyotime Biotechnology). Coverslips were mounted with D.P.X. (Mounting Medium; Sigma-Aldrich), and the tissues were scanned using “Pannoramic SCAN” (3DHISTECH, Budapest, Hungary).

### Immunofluorescence staining

Paraffin sections were prepared as described above for HE staining. The slides were deparaffinized and processed for antigen retrieval with citrate-EDTA antigen retrieval solution (Beyotime Biotechnology). The slides were then blocked with 5% BSA for 30 min and incubated overnight at 4 °C with primary antibodies. Following three washes with PBST, the slides were incubated for 30 min with secondary antibodies. Then the slides were washed and stained with DAPI (2 µg/ml, Abcam, Shanghai, China). Coverslips were mounted with “Antifade Mounting Medium” (Beyotime Biotechnology), and the tissues were scanned using “Pannoramic SCAN” (3DHISTECH).

### Statistical analysis

Statistical significance was determined by the Mann–Whitney U test unless otherwise specified. ELISA measurements were performed in triplicate. Multiple values were used to calculate the mean, the standard deviation or the standard error, as outlined in the respective figure legends. *p*-values <0.05 (*) were considered significant. Statistical analysis was conducted using SPSS software (IBM, New York, USA).

### Supplementary information

Supplement

Original WB figures

aj-checklist

## Data Availability

All data generated or analyzed during this study are available from the corresponding author upon reasonable request.
