# Supplementary material for: Interfering with Rac1-activation during neonatal monocyte-macrophage differentiation influences the inflammatory responses of M1 macrophages
Source: Cell Death Dis. 2023 Sep 21;14(9):619. doi: 10.1038/s41419-023-06150-y (PMC10514032; doi:10.1038/s41419-023-06150-y)
Supplement: Supplementary file 1 — Supplement [file 41419_2023_6150_MOESM1_ESM.pdf]

Supplement

Supplement Table 1. Antibodies used in the experiments.

Antigen	Clone	Class	Source	Company	Order no.	Stain	[a]Assay
GAPDH	D4C6R	IgG1	mouse	Cell Signaling	97166S		WB
YB1		IgG	rabbit	Proteintech	20339-1-AP		WB/FACS/ RNA-IP
Phospho-YB1		IgG	rabbit	Cell signaling	2900S		WB
Rac1	4A4B11	IgG2b	mouse	Proteintech	66122-1-Ig		WB
PABPC1		IgG	rabbit	Proteintech	10970-1-AP		WB/RNA-IP
PABPC4		IgG	rabbit	Proteintech	14960-1-AP		WB
MYD88	2E10D3	IgG2b	mouse	Proteintech	67969-1-Ig		WB
IRAK4	1A4C4	IgG2a	mouse	Proteintech	67180-1-Ig		WB
β-tubulin	1D4A4	IgG2a	mouse	Proteintech	66240-1-Ig		WB
TRAF6		IgG	rabbit	Bioss Antibodies	bs-2830R		WB
CD68	ED1	IgG1	mouse	Bio-Rad	MCA341B	Biotin	CI
anti-mouse IgG			goat	Proteintech	SA00001-1	HRP	WB
anti-rabbit IgG			donkey	Proteintech	SA00001-9	HRP	WB
isotype		IgG	rabbit	Proteintech	30000-0-AP		RNA-IP
Rac1			Mouse	Cell Signaling	8815S (8631)		PD
CD64	10.1	IgG1	Mouse	Biolegend	305006	FITC	FACS
CD80	2D10	IgG1	mouse	Biolegend	305231	PerCP/Cy5.5	FACS

CD86	BU63	IgG1	mouse	Biolegend	374215	PerCP/Cy5.5	FACS
HAL-DR	L243	IgG2a	mouse	Proteintech	CL488-65218	CoraLite Plus 488	FACS
TLR4	HTA125	IgG2a	mouse	Biolegend	312805	PE	FACS
IL-6	MQ2-13A5	IgG1	rat	Biolegend	501106	PE	FACS
TNF-α	MAb11	IgG1	mouse	Biolegend	502923	PerCP	FACS
anti-rabbit IgG	Poly4064	Polyclonal Ig	donkey	Biolegend	406403	FITC	FACS
CD68	ED1	IgG1	mouse	Abcam	ab31630		IF
CD86		IgG	rabbit	Proteintech	13395-1-AP		IF
CD206		IgG	rabbit	Proteintech	18704-1-AP		IF
anti-mouse IgG		IgG	goat	Invitrogen	31660	Rhodamine	IF
anti-rabbit IgG		IgG	goat	Abcam	ab7086	FITC	IF

a - WB, Western blot; FACS, fluorescence-activated cell-sorting; RNA-IP, RNA-immunoprecipitation; PD, pull-down-assay; IF: immunofluorescence; CI: Cell isolation.

Supplement Table 2. Primers and siRNA used in the experiments.

name	Forward primer Sequence (5'->3')	Reverse primer Sequence (5'->3')
human HPRT	CCTGGCGTCGTGATTAGTGAT	AGACGTTCAGTCCTGTCCATAA
human IL-1β	GAAATGATGGCTTATTACAGTGGC	TAGTGGTGGTCGGAGATTCG
human IL-6	ACTCACCTCTTCAGAACGAATTG	CCATCTTTGGAAGGTTCAGGTTG
human YB-1	ACAAGAAGGTCATCGCAACG	CTCTCCATCTCCTACACTGCG
human PABPC1	CAGGCTCACCTCACTAACCAG	GGTAGGGGTTGATTACAGGGT
human PABPC4	AAACACGAGGATGCCAATAAGG	TGCCTGCCGTTCTACTTTCTT

human IRAK4	CCTGACTCCTCAAGTCCAGAA	ACAGAAATGGGTCGTTCATCAAA
human TRAF6	TTTGCTCTTATGGATTGTCCCC	CATTGATGCAGCACAGTTGTC
human MYD88	CTGAGCGTTTCGATGCCTTC	GCGGTCAGACACACACAACT
human TLR4	AGACCTGTCCCTGAACCCTAT	CGATGGACTTCTAAACCAGCCA
human GAPDH	ACAACTTTGGTATCGTGGAAGG	GCCATCACGCCACAGTTTC
human IGF2BP1	CCTTTATGCAGGCTCCCGA	TTCGGGTGGTGCAATCTTGA
NC siRNA	UUCUCCGAACGUGUCACGUTT	ACGUGACACGUUCGGAGAATT
YB1 siRNA 1	GGAGGCAGCAAAUGUUACATT	UGUAACAUUUGCUGCCUCCTT
YB1 siRNA 2	CCACGCAAUUACCAGCAAATT	UUUGCUGGUAAUUGCGUGGTT

Supplement Table 3. Down- or up-regulated protein in the *in vitro* differentiated neonatal macrophages (day 6) derived in the presence of NSC23766 (B), as compared to macrophages derived in the absence of NSC23766 (A).[a]

[b]Protein	Gene	[c]$Mean_{B/A}$	[d]$Lg2_{MeanB/A}$	[e]P $value_{B/A}$	upregulation / downregulation
X5D2U3	STK39	0.646	-0.630	0.021	down
Q7RTV0	PHF5A	0.637	-0.650	0.043	down
Q9BZE9	ASPSCR1	0.635	-0.654	0.018	down
Q13011	ECH1	0.623	-0.683	0.028	down
D6W5Y5	CIRBP	0.615	-0.702	0.047	down
Q6UXH1	CRELD2	0.610	-0.714	0.041	down
P00918	CA2	0.597	-0.743	0.036	down
P17050	NAGA	0.581	-0.783	0.049	down
A0A024R3C3	CHORDC1	0.581	-0.784	0.038	down
B4E0K9		0.569	-0.814	0.020	down
Q07954	LRP1	0.568	-0.816	0.008	down
Q8NBJ5	COLGALT1	0.567	-0.819	0.022	down
P67809	YBX1	0.563	-0.829	0.029	down
Q597H1	TRG14	0.552	-0.857	0.021	down
P17676	CEBPB	0.552	-0.857	0.007	down
B4DPN0		0.547	-0.871	0.009	down
A0A8C8KJP3	CRELD1	0.546	-0.873	0.031	down
B2R888		0.537	-0.897	0.012	down
P02649	APOE	0.537	-0.897	0.014	down
Q86U75		0.532	-0.912	0.023	down
J3KS15	MRPL58	0.530	-0.917	0.026	down
A6XND9		0.524	-0.932	0.024	down
O75380	NDUFS6	0.516	-0.954	0.019	down
P54802	NAGLU	0.514	-0.960	0.003	down
A0A5F9ZHR7	ACP2	0.510	-0.972	0.003	down

A4D1V4	MRPL32	0.503	-0.990	0.003	down
M0QYI1	PNKP	0.501	-0.998	0.016	down
P55072	VCP	0.498	-1.007	0.004	down
A3KPE2	APOC3	0.496	-1.012	0.018	down
Q03405	PLAUR	0.483	-1.050	0.017	down
B4DJQ8		0.483	-1.051	0.016	down
B4DYX2		0.482	-1.052	0.038	down
O60568	PLOD3	0.467	-1.098	0.029	down
P62861	FAU	0.448	-1.160	0.041	down
Q02809	PLOD1	0.411	-1.282	0.017	down
Q969X0	RILPL2	0.400	-1.324	0.033	down
Q8WUA7	TBC1D22A	0.388	-1.367	0.001	down
A0A384MDQ7		0.357	-1.485	0.015	down
Q9BUT1	BDH2	0.332	-1.592	0.022	down
B3KP88		0.306	-1.709	0.023	down
Q9HD89	RETN	0.302	-1.725	0.012	down
A0A024QYX3	RBM3	0.266	-1.913	0.008	down
A0A0G2JS82	AP2A2	0.194	-2.365	0.002	down
F2YQ21		0.121	-3.043	0.010	down
O95487	SEC24B	6.292	2.654	0.037	up
Q2M389	WASHC4	4.605	2.203	0.026	up
E9PC47	LIMK1	4.000	2.000	0.017	up
A0A6Q8PHE5	UBE3A	3.173	1.666	0.003	up
Q9Y5K6	CD2AP	3.106	1.635	0.015	up
Q86WA9	SLC26A11	2.950	1.561	0.014	up
Q9BTU6	PI4K2A	2.910	1.541	0.032	up
Q8IWR0	ZC3H7A	2.825	1.498	0.008	up
Q9P0I2	EMC3	2.788	1.479	0.015	up
B4DLM2		2.787	1.479	0.008	up
Q01432	AMPD3	2.762	1.466	0.050	up
J9R021	eIF3a	2.583	1.369	0.026	up
A0A140VJP8		2.442	1.288	0.015	up
Q32P41	TRMT5	2.442	1.288	0.008	up
B4DLR8	NQO1	2.440	1.287	0.006	up
P50749	RASSF2	2.419	1.275	0.002	up
Q6ICG6	KIAA0930	2.393	1.259	0.026	up
P22681	CBL	2.378	1.250	0.009	up
Q86T03	PIP4P1	2.346	1.230	0.025	up
D6RGX2	UFSP2	2.323	1.216	0.018	up
Q13825	AUH	2.268	1.181	0.050	up
C9JF17	APOD	2.252	1.171	0.012	up
O14617	AP3D1	2.188	1.130	0.007	up
Q9C0C9	UBE2O	2.162	1.113	0.014	up
Q5U003	CCR1	2.159	1.110	0.033	up
Q9Y666	SLC12A7	2.102	1.072	0.014	up
O75179	ANKRD17	2.078	1.055	0.018	up
A0A0A0MRA8	EPB41L3	2.074	1.052	0.013	up
A0JNV7	EMR2	2.071	1.050	0.032	up
Q8N8A2	ANKRD44	2.070	1.050	0.008	up

Q9NQX3	GPHN	2.040	1.028	0.035	up
A0A024R8V0		2.037	1.027	0.028	up
O14545	TRAFD1	2.016	1.012	0.047	up
P50416	CPT1A	1.982	0.987	0.044	up
A0A7I2YQL3	WDR26	1.979	0.985	0.050	up
Q96C23	GALM	1.968	0.976	0.040	up
P59768	GNG2	1.955	0.967	0.028	up
Q8NFX3		1.928	0.947	0.035	up
O00160	MYO1F	1.915	0.937	0.043	up
A0MNP2	WDR57	1.907	0.931	0.013	up
O00505	KPNA3	1.854	0.891	0.021	up
Q9Y295	DRG1	1.851	0.888	0.021	up
A0A8I5KRG1	DCAF7	1.838	0.878	0.028	up
C9J975	AGAP3	1.828	0.870	0.007	up
Q58F09	GCS1	1.828	0.870	0.026	up
Q9UPN7	PPP6R1	1.826	0.869	0.005	up
Q9Y385	UBE2J1	1.799	0.848	0.011	up
Q9UH65	SWAP70	1.795	0.844	0.035	up
O60268	KIAA0513	1.786	0.837	0.046	up
B4DV79	EIF3B	1.772	0.826	0.032	up
Q8TDX7	NEK7	1.764	0.819	0.043	up
Q14203	DCTN1	1.763	0.818	0.046	up
Q96JH7	VCPIP1	1.757	0.814	0.032	up
P59665	DEFA1	1.753	0.810	0.035	up
Q92734	TFG	1.735	0.795	0.021	up
O43252	PAPSS1	1.733	0.793	0.024	up
A0A0H4Q180		1.718	0.780	0.032	up
Q9Y286	SIGLEC7	1.692	0.759	0.030	up
A0A7P0T8D4	SNX9	1.686	0.754	0.008	up
Q9H0C8	ILKAP	1.677	0.746	0.021	up
Q6NZ61	RHEB	1.663	0.734	0.038	up
Q9BW34	EEF1D	1.661	0.732	0.014	up
P55263	ADK	1.647	0.720	0.017	up
B3KMH8		1.646	0.719	0.009	up
Q9Y2U5	MAP3K2	1.644	0.717	0.003	up
Q6NS36	FTH1	1.635	0.710	0.046	up
Q9BRF8	CPPED1	1.601	0.679	0.019	up
P04181	OAT	1.595	0.673	0.004	up
Q9Y4E6	WDR7	1.593	0.672	0.019	up
Q9Y383	LUC7L2	1.578	0.658	0.032	up
Q96T51	RUFY1	1.563	0.644	0.028	up
Q13045	FLII	1.538	0.621	0.006	up
F8W9X7	CCDC93	1.532	0.616	0.041	up
K7EIU8	SMAD4	1.523	0.607	0.015	up
P00488	F13A1	1.515	0.599	0.007	up
B4DWW8		1.510	0.595	0.014	up
Q6GV32		1.504	0.588	0.003	up
O43815	STRN	1.503	0.587	0.048	up
A0A2P0CTB1	PRXL2B	2.516	1.331	0.016	up

Q9ULH1	ASAP1	2.033	1.023	0.024	up
Q495P7	TLR8	1.892	0.920	0.038	up
A0A7P0T8J7	AAR2	1.861	0.896	0.044	up
H0YI33	ARHGAP9	1.853	0.890	0.002	up
A8K586		1.641	0.714	0.029	up
Q12974	PTP4A2	1.620	0.696	0.019	up
A0A024QZ03	CCDC22	1.547	0.630	0.019	up

a - The macrophages were lysed with DB lysis buffer (8 M Urea, 100 mM TEAB, pH 8.5). The LC-MS analyses were performed using an EASY-nLCTM 1200 UHPLC system (Thermo Fisher) coupled with a Q ExactiveTM HF-X mass spectrometer (Thermo Fisher) in Novogene Co., Ltd. (Beijing, China). The normalized data of in vitro differentiated macrophages (day 6) in the presence (B) or absence (A) of NSC23766 are listed.

b - UniProt ID were listed in this column.

c - In order to define the down- or upregulation of the respective proteins, the expression of the proteins in the presence of NSC23766 (B) was divided by the expression in the absence of NSC23766 (A). The mean of this ratios of three samples were calculated.

d - The Lg2 of $mean_{B/A}$ was calculated.

e - To determine the significance of the differences between group A and B. Student's t-test was performed and the P value was calculated.

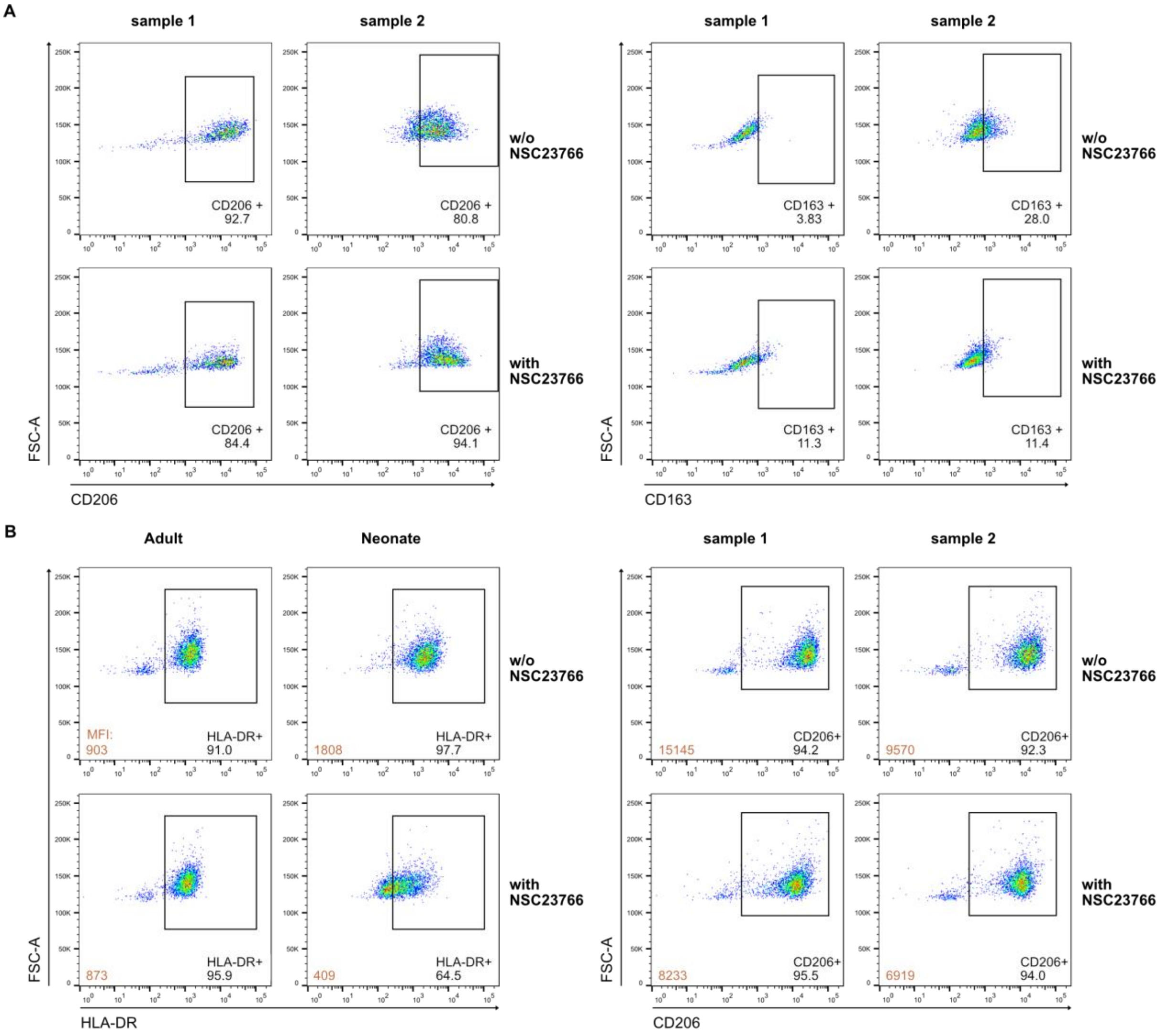

Figure S1. Surface marker expression in neonatal and adult macrophages.

(A) Dot plots show inconsistent expression of surface markers (M2 macrophages) of the in vitro differentiated M1 macrophages on day 6 with or without NSC23766. Macrophages were collected on day 6, then stained with anti-CD206 and CD163 antibodies. Expression of these markers was assessed in flow cytometry. Numbers indicate percentages of protein-expressing cells, respectively.

(B) Representative dot plots show differential expression of HLA-DR and CD206 of the in vitro differentiated M1 macrophages from neonates and adults with or without NSC23766. Macrophages were collected on day 6, then stained with anti-HLA-DR and CD206 antibodies. Expression of surface markers were assessed in flow cytometry. Numbers in the bottom left indicate mean fluorescence intensity (MFI) of HLA-DR or CD206 in cells, respectively. Numbers in the bottom right indicate percentages of protein-expressing cells, respectively.

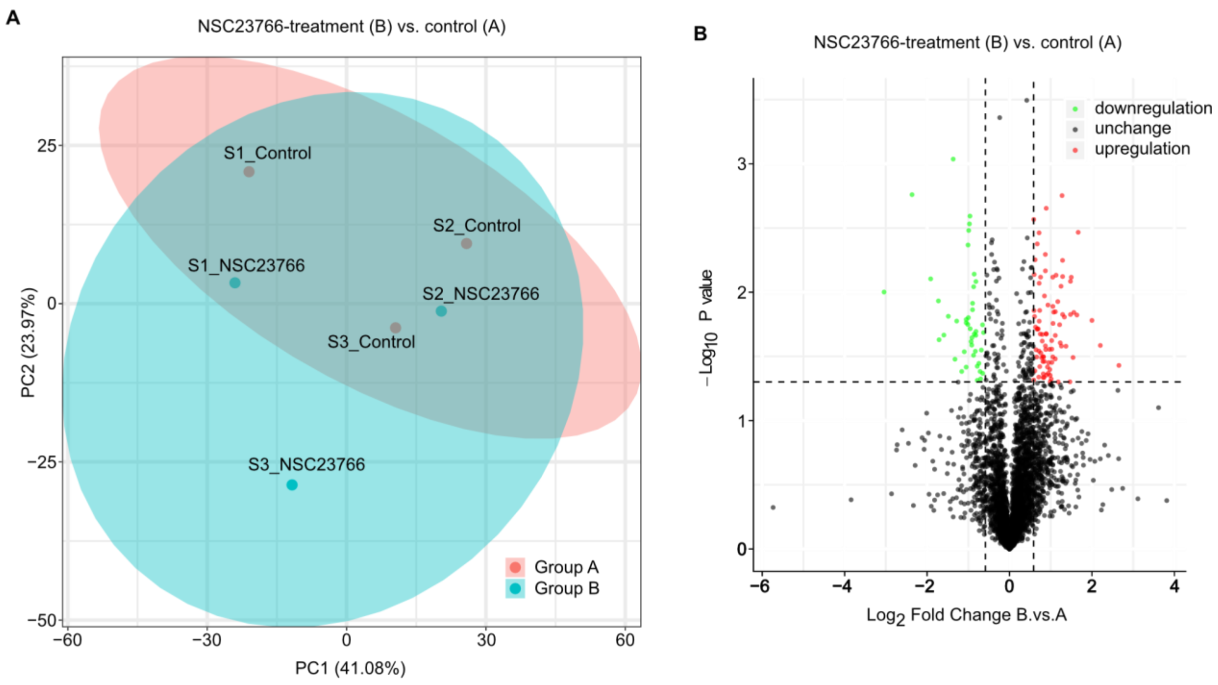

Figure S2. Information of the LC-MS experiment.

(A) Neonatal monocytes from umbilical cord blood were in vitro differentiated into M1 macrophages with (B) or without (A) NSC23766. Cells were collected and lysed on day 6. Cell lysates were analyzed in label free mass spectrometry in three independent experiments (S1, S2 and S3) and the principal component analysis (PCA) were performed in python (3.5.0).

(B) The differential expression of proteins was analyzed in R (R-3.4.3). The volcano plot was generated and proteins were presented in this plot according to the comparison of M1 macrophages with NSC23766 (A) to the control cells without NSC23766 (B). Green dots, proteins downregulated; Red dots, proteins upregulated; black dots, proteins not significantly altered.

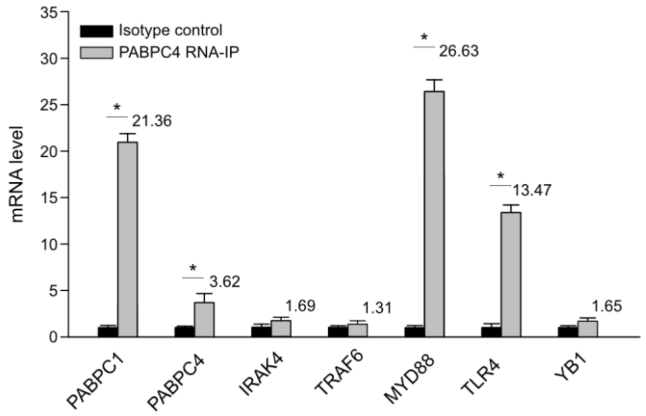

Figure S3. Results of RNA-IP for anti-PABPC4. Neonatal monocytes from umbilical cord blood were in vitro differentiated into M1 macrophages. Cells were collected and lysed on day 6. RNA-IP was performed using isotype control (black columns) or anti-PABPC4 antibody (gray columns). Numbers above the column indicate fold change of PABPC4-binding RNA to isotype control.
