# Supplementary material for: Interfering with Rac1-activation during neonatal monocyte-macrophage differentiation influences the inflammatory responses of M1 macrophages
Source: Cell Death Dis. 2023 Sep 21;14(9):619. doi: 10.1038/s41419-023-06150-y (PMC10514032; doi:10.1038/s41419-023-06150-y)

Orginal Western blot pictures

Figure 1E

Rac1: 21 KD

GAPDH: 37 KD

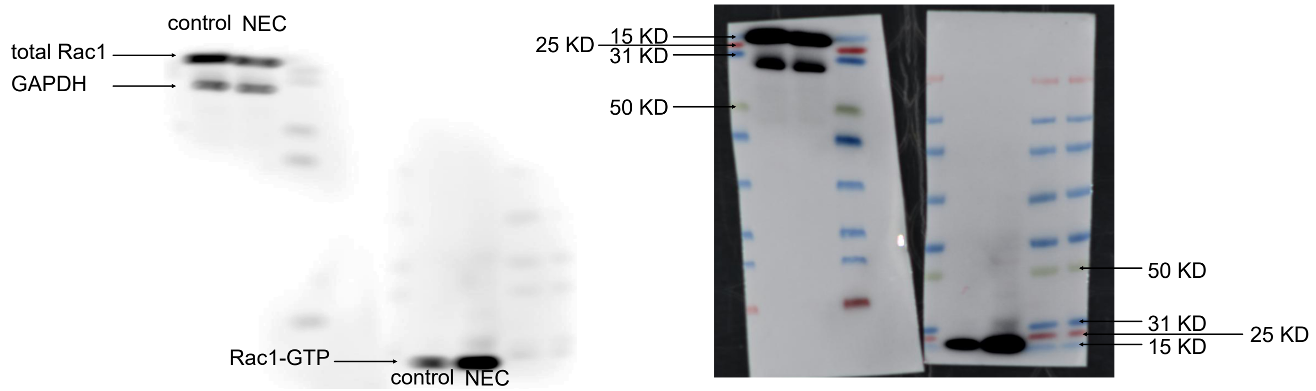

Figure 2A

Rac1: 21 KD

GAPDH: 37 KD

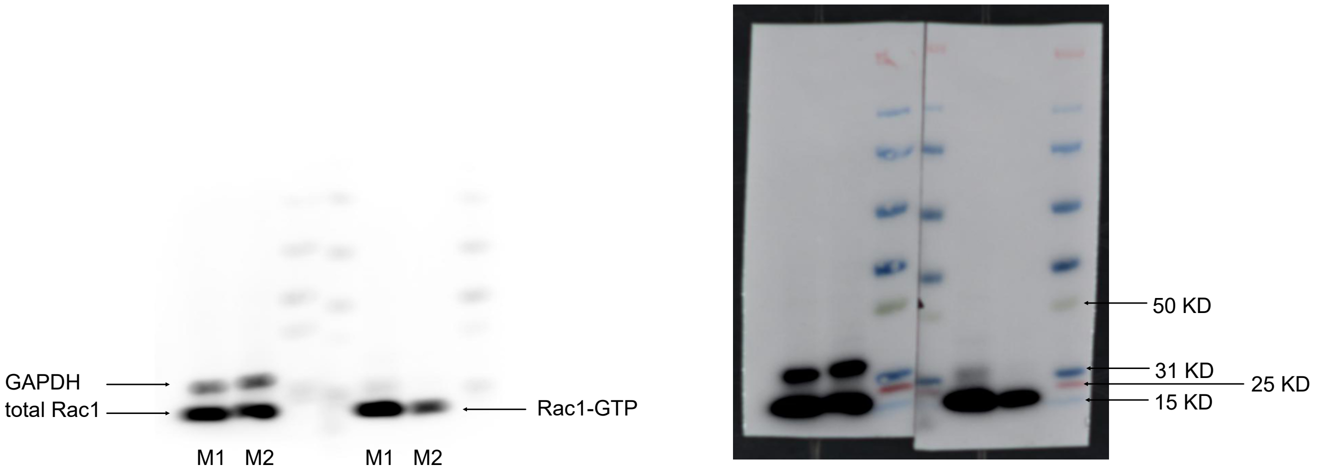

Figure 3C

p-YB1: 49 KD

YB1: 49 KD

GAPDH: 37 KD

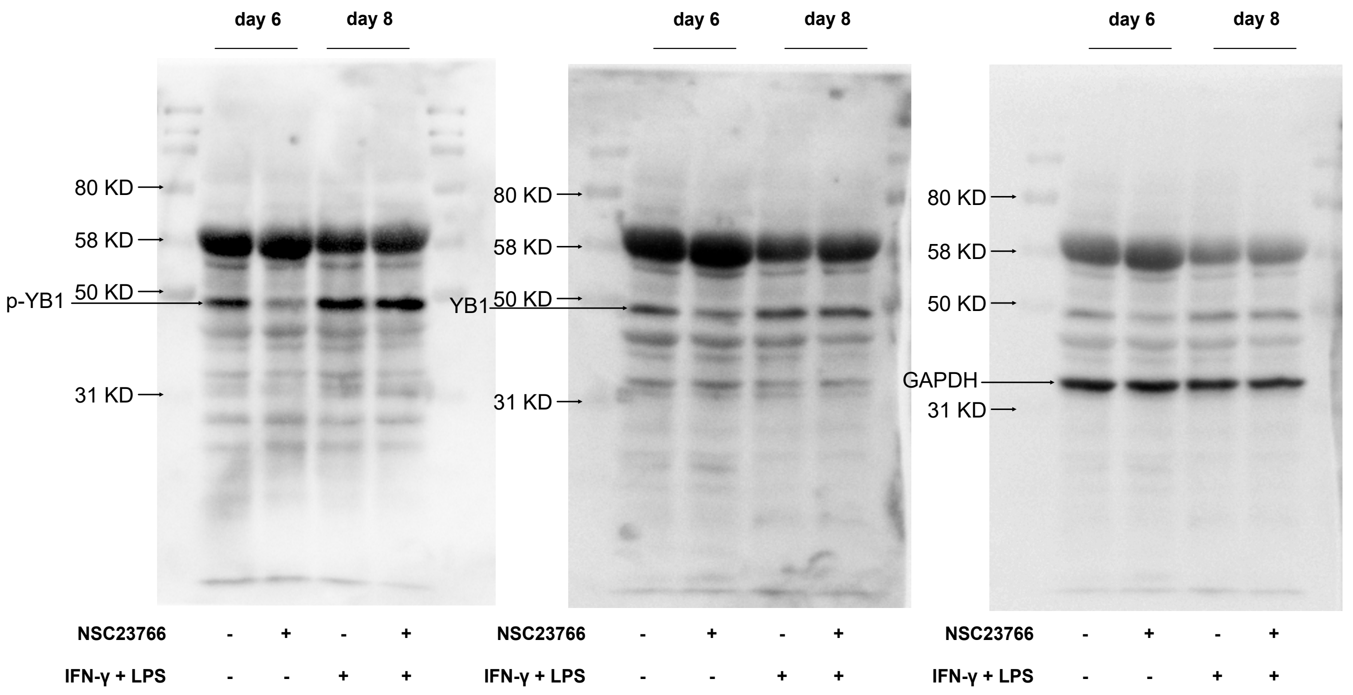

Figure 5C

PABPC1: 71 KD PABPC4: 70 KD p-YB1: 49 KD YB1: 49 KD

GAPDH: 37 KD

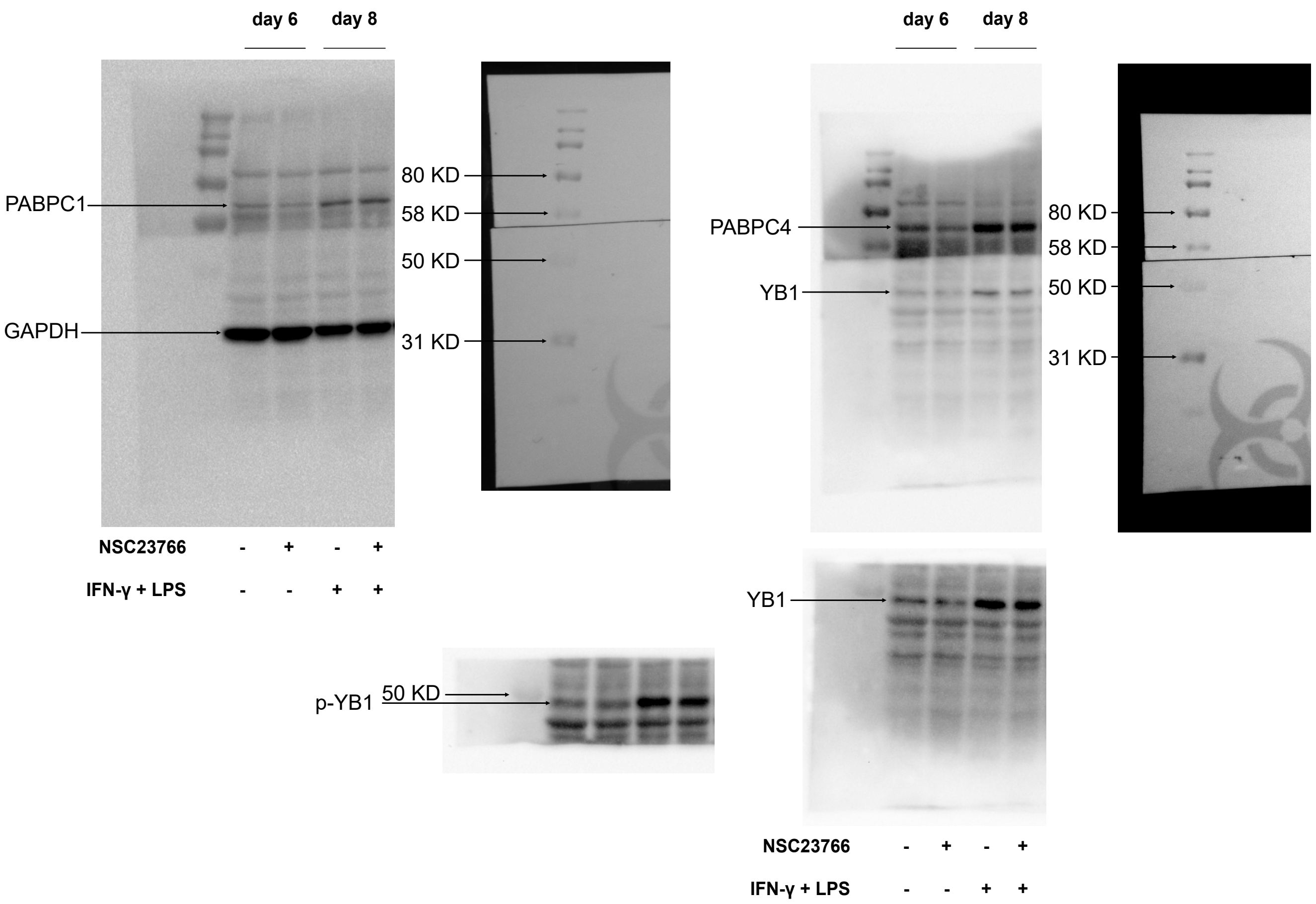

Figure 6D

IRAK4: 52 KD TRAF6: 60 KD MYD88: 33 KD

GAPDH: 37 KD β-Tubulin: 51 KD

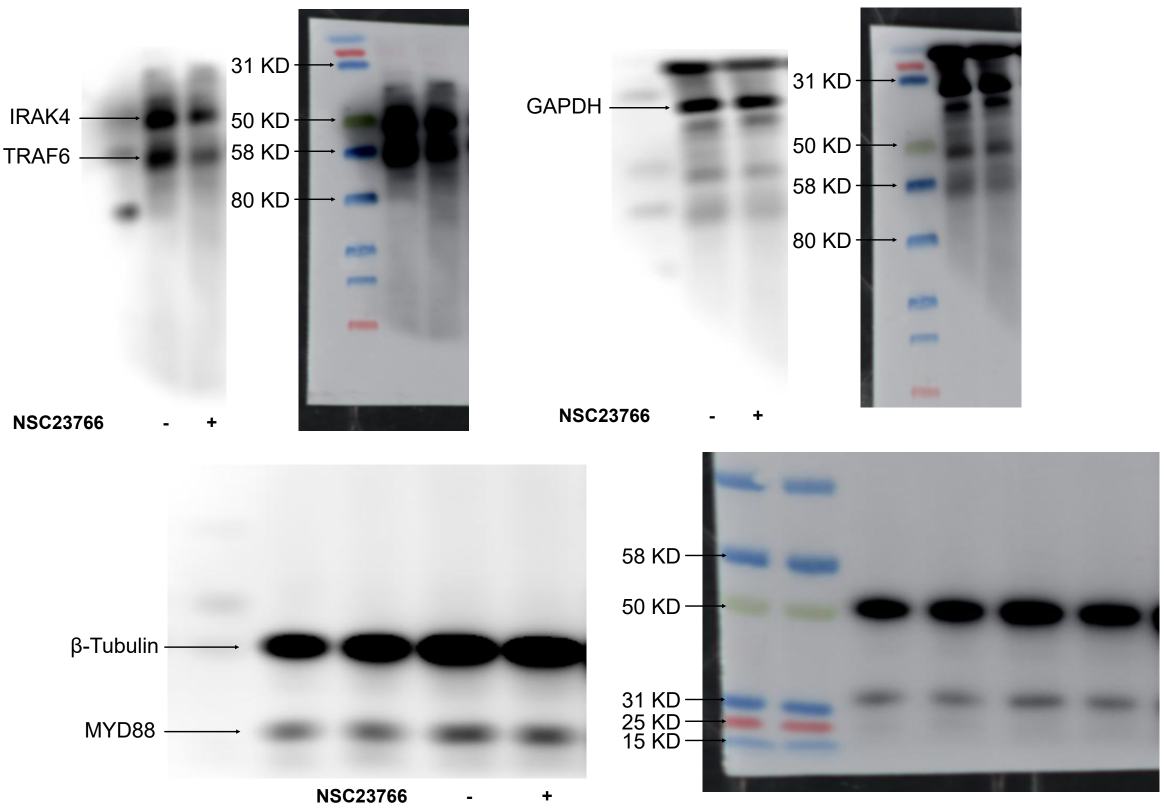

Figure 6H

YB1: 49 KD

GAPDH: 37 KD

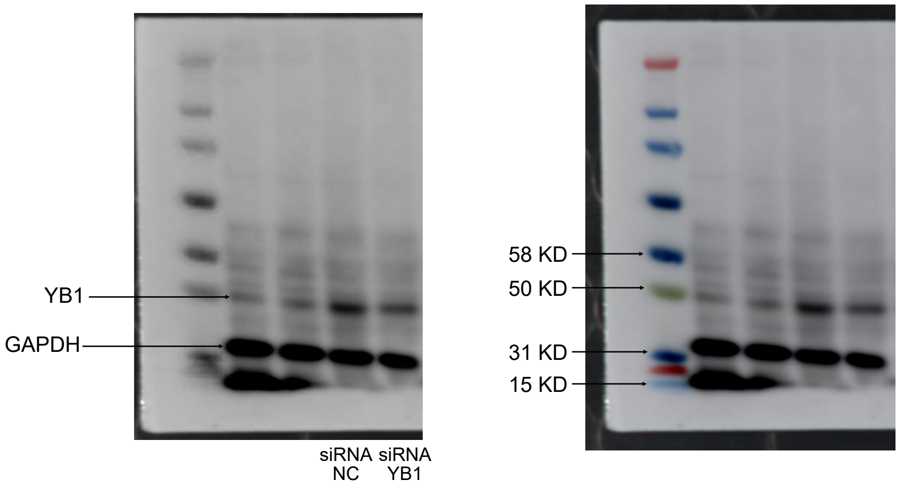

Figure 6D

IRAK4: 52 KD

TRAF6: 60 KD

MYD88: 33 KD

GAPDH: 37 KD

β-Tubulin: 51 KD

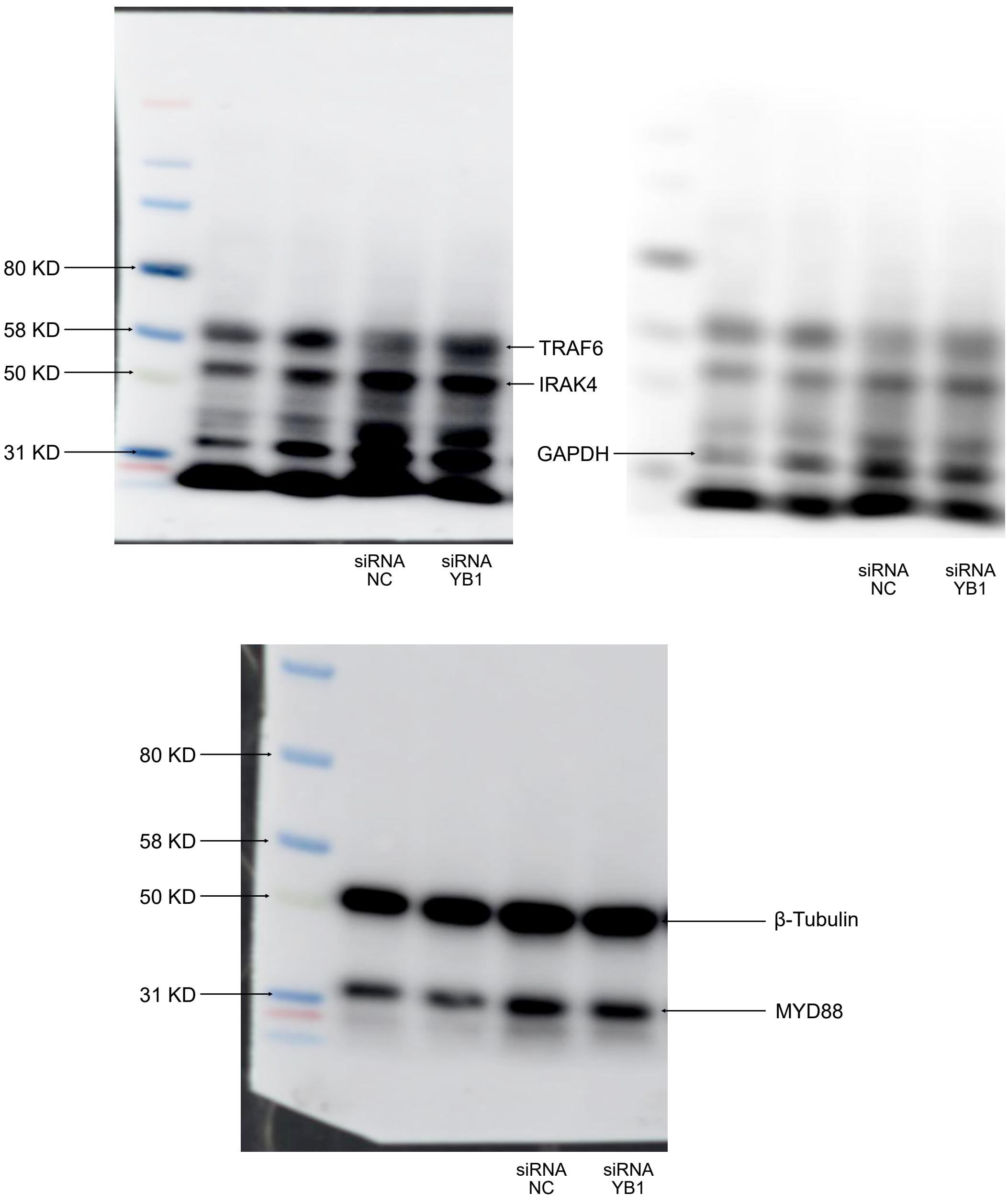

Supplement: Supplementary file 2 — Original WB figures [file 41419_2023_6150_MOESM2_ESM.pdf]
